# Dendrobium Nobile Alcohol Extract Extends the Lifespan of *Caenorhabditis elegans* via *hsf-1* and *daf-16*

**DOI:** 10.3390/molecules29040908

**Published:** 2024-02-19

**Authors:** Linfeng Li, Zhen Liu, Huiling Hu, Renming Cai, Jingdou Bi, Qin Wang, Xiaogang Zhou, Huairong Luo, Chun Zhang, Runlan Wan

**Affiliations:** 1 Department of Oncology, The Affiliated Hospital of Southwest Medical University, Luzhou 646000, China; 20210599120119@stu.swmu.edu.cn; 2 School of Pharmacy, Southwest Medical University, Luzhou 646000, China20210599120062@stu.swmu.edu.cn (H.H.); 20210599120004@stu.swmu.edu.cn (R.C.); wq_ring@hotmail.com (Q.W.);; 3 Key Laboratory of Luzhou City for Aging Medicine, Department of Pharmacology, School of Pharmacy, Southwest Medical University, Luzhou 646000, China; lhr@swmu.edu.cn; 4 Key Laboratory of Bio-Resource and Eco-Environment of Ministry of Education, College of Life Sciences, Sichuan University, Chengdu 610065, China; 18435178496@163.com; 5 Dazhou Vocational College of Chinese Medicine, Dazhou 635000, China; 6 Ministry of Education & Medical Electrophysiological Key Laboratory of Sichuan Province, Institute of Cardiovascular Research, Southwest Medical University, Luzhou 646000, China

**Keywords:** *Caenorhabditis elegans*, dendrobium nobile, antiaging, DAF-16, alzheimer’s disease, parkinson’s disease

## Abstract

*Dendrobium nobile* is a traditional Chinese herb with anti-inflammatory, antioxidant, and neuroprotective properties. However, its antiaging effects are unclear. Herein, we studied the aging-related functions and the mechanism of action of the alcohol extract of *Dendrobium nobile* (DnAE) in the model organism *Caenorhabditis elegans*. The results indicated that 1 mg/mL DnAE slowed lipofuscin accumulation, decreased the levels of reactive oxygen species, elevated superoxide dismutase activity, enhanced oxidative and heat stress resistance, extended the lifespan of nematodes, protected their dopamine neurons from 6-hydroxydopamine-induced neurodegeneration, and reduced Aβ-induced neurotoxicity. DnAE upregulated the mRNA expression of the transcription factors DAF-16 and HSF-1, promoted the nuclear localization of DAF-16, and enhanced the fluorescence intensity of HSP-16.2. However, it had no effect on the lifespan of DAF-16 mutants. Thus, DnAE can significantly extend lifespan, enhance heat stress tolerance, and delay age-related diseases through a DAF-16-dependent pathway.

## 1. Introduction

Aging is a natural process characterized by structural deterioration, loss of bodily functions, and decreased immunity [1]. Various studies have shown that aging is a significant risk factor for degenerative diseases, including cardiovascular [2] and neurodegenerative diseases, such as Alzheimer’s disease (AD) and Parkinson’s disease (PD) [3]. Natural active ingredients are widely used in antiaging research due to their versatile structures and multitarget binding. For instance, resveratrol is a recognized natural active ingredient in the antiaging process that can extend the lifespan of mice through caloric restriction [4]. Blueberry extract has been shown to promote the lifespan and stress tolerance of *Caenorhabditis elegans* (*C. elegans*) via DAF-16 [5], and ginsenosides extend the lifespan of *C. elegans* and reduce the accumulation of lipofuscin [6], a complex of oxidized lipids and proteins that accumulates during cellular and tissue senescence that is often considered a marker of senescence in *C. elegans* [7]. Given the increasing global aging problem, developing natural products that can delay aging or promote a healthy lifespan is crucial.

*Dendrobium nobile* Lindl is an epiphytic herb of the genus *Dendrobium* from the family Orchidaceae. It is traditionally used in Chinese medicine to nourish yin, clear heat, and promote overall health [8]. Modern research has highlighted *D. nobile’s* significant biological effects in reducing cancer risk [9], preventing diabetes [10], improving oxidative stress [11], and preventing neurodegenerative diseases such as AD and PD [12]. It mainly consists of alkaloids, polysaccharides, and phenolic compounds. Studies have demonstrated that *D. nobile* alkaloids exert beneficial effects on glucose and lipid metabolism in mouse livers [13], attenuate LPS-induced tau protein hyperphosphorylation in rat hippocampi, and prevent LPS-induced apoptosis in rat brains [14]. Its phenolic compounds exhibit anti-inflammatory activity by targeting various inflammation-associated cytokines [15]. Synergistic interactions between multiple compounds from the same plant can provide enhanced biological activity [16]. Our previous results showed that the alcohol extract of *D. nobile* (DnAE) could exert immune-enhancing, antioxidant, and antiaging effects by remodeling the intestinal microecosystem and downregulating the expression of proaging genes in mouse brains [17]. Therefore, we obtained the total extract of *D. nobile* using alcohol as the most suitable solvent and investigated whether the extract could delay aging and prevent age-related diseases.

*C. elegans* is a popular model for studying stress resistance and longevity due to its short lifespan, mature genetic pathways, and high reproductive rate [18]. Stress resistance and aging in *C. elegans* are regulated by multiple signaling pathways including the insulin/insulin-like growth factor (IIS) pathway, mitogen-activated protein kinase pathway, diet restriction pathway, germline pathway, and key transcription factors such as SKN-1 [19]. The IIS is highly evolutionarily conserved and is the first pathway found to significantly affect the longevity of various species. Reduced IIS increases cellular tolerance to a variety of stresses, thus prolonging lifespan [20]. DAF-16 and HSF-1 are the two key transcription factors of the IIS pathway in *C. elegans*. DAF-16 is the most extensively studied factor to prolong the lifespan of *C. elegans*, one important mechanism of which is that DAF-16 reduces reactive oxygen species (ROS) levels and increases the activities of antioxidant enzymes such as superoxide dismutase (SOD), glutathione S-transferase (GST), and catalase by regulating the downstream genes *sod-3*, *gst-4*, and *ctl-1* [21,22,23]. HSF-1 is involved in modulating the lifespan and longevity pathways of *C. elegans* by regulating the expression of various stress proteins such as heat shock proteins (HSPs) [24].

In the present study, we investigated the antiaging performance of DnAE and its molecular mechanism by detecting the vitality and antioxidant biomarkers of *C. elegans*. Furthermore, we identified that DnAE is neuroprotective and delays nematode aging by activating the stress-related transcription factors DAF16/FOXO and HSF-1 from the IIS pathway. These findings provide new insights into the potential protective role of *D. nobile* in healthy aging and aging-related diseases.

## 2. Results

### 2.1. Chemical Composition of DnAE

**Figure 1:**
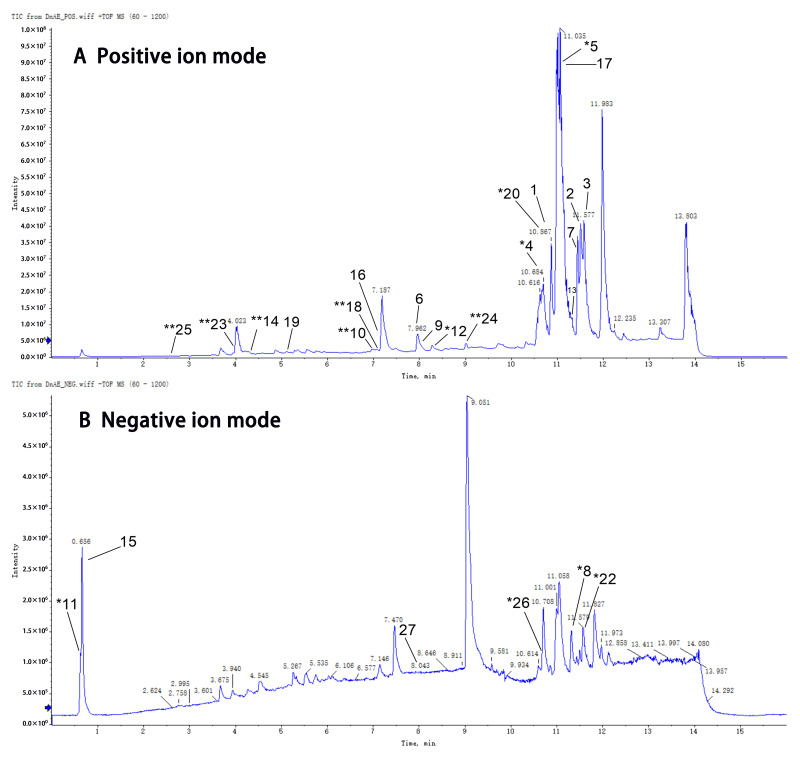
Total ion chromatograms of DnAE in positive ion mode (**A**) and negative ion mode (**B**). * Confirmed with standards. ** Tentatively identified based on library and/or literature data. The bold numbers: indicate the corresponding numbers in Table 1.

The composition characteristics of DnAE were determined by UHPLC-Q-TOF-MS/MS in both positive and negative ion modes (Figure 1). The compounds were initially identified by comparisons using the Shanghai Applied Protein Technology database and public databases Mass Bank, Metlin, HMDB, and MoNA and subsequently confirmed by standards and the literature (Figure S1). As shown in Table 1, DnAE was mainly composed of 27 known constituents, which accounted for 95% of the total peaks observed and included palmitic amide, glyceryl monostearate, linoleic acid, mubironine B, quinic acid, dendroside G, and dendrobine. Eight components were qualified by the standards, and the remaining components were tentatively identified based on the library and/or literature data.

### 2.2. DnAE Increases the Lifespan and Improves the Health Span of C. elegans

**Figure 2:**
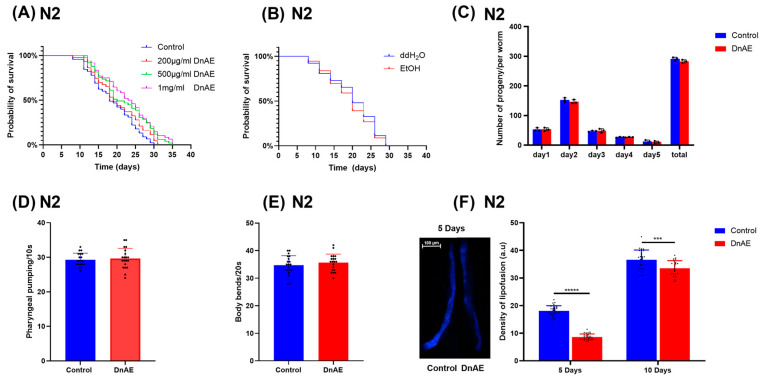
Effect of DnAE on the lifespan and healthspan of wild-type *C. elegans* (N2). (**A**) Survival of N2 worms treated with or without different concentrations of DnAE. *n* ≥ 60. (**B**) Survival of N2 worms treated with EtOH (214 µmol/mL, consistent with the concentration of alcohol contained in 1 mg/mL DnAE) or ddH_2_O. *n* ≥ 60. (**C**) Average and total number of eggs produced per hermaphroditic worm. *n* ≥ 20. (**D**) Number of pharyngeal pumping events by worms. (**E**) Number of bending events in worms after 20 s. *n* ≥ 20. (**F**) Representative fluorescence images show lipofuscin levels in N2 after 5 days. The bar chart indicates the fluorescence intensity of N2 treated with DnAE for 5 and 10 days. (*n* ≥ 20, *** *p* < 0.001, ***** *p* < 0.00001, *t* test).

To investigate the effects of DnAE on the lifespan of *C. elegans* and determine the optimal dosage, we treated *C. elegans* with different DnAE concentrations. DnAE significantly extended lifespan, with the best effect observed at a concentration of 1 mg/mL, which increased the lifespan by up to 21.37% (*p* = 0.0013) (Figure 2A and Table S1). A control experiment was conducted using an equivalent alcohol concentration to exclude the effects of solvents on the experimental results. The lifespan of *C. elegans* was not significantly different between ddH_2_O treatment and alcohol treatment (Figure 2B and Table S2). The reproductive toxicity of a drug can be reflected by the number of eggs produced by the worm [32]. A comparable number of eggs were produced by the treatment and control groups (Figure 2C and Table S3), indicating the reproductive safety of DnAE. Dietary restriction and muscle activity can also affect the lifespan of worms [33]. Supplementation with DnAE caused an imperceptible change in the number of bends and swallows of *C. elegans* within a certain timeframe (Figure 2D,E and Tables S4 and S5). The level of lipofuscin is a commonly used indicator of aging in *C. elegans* [34]. We used the spontaneous fluorescence intensity of lipofuscin to measure its content in the worms. The results revealed increasing lipofuscin levels with increasing age (Figure 2F and Table S6). We measured the fluorescence levels of *C. elegans* treated with or without DnAE treatment on the 5th and 10th days and found that 1 mg/mL DnAE significantly reduced the increase in lipofuscin. The difference was most significant on the 5th day but decreased over time, consistent with the results showing an increase in the lifespan of *C. elegans* (Figure 2F).

### 2.3. DnAE Increased Heat Stress Tolerance by Upregulating the Expression of hsf-1 and HSPs

**Figure 3:**
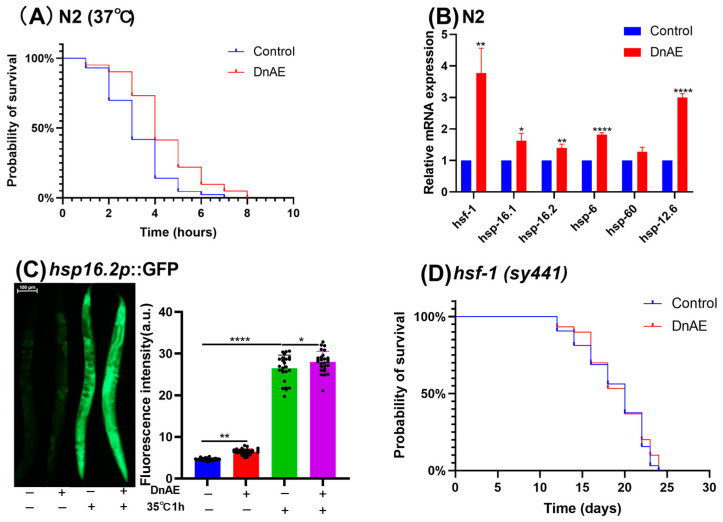
DnAE increased heat stress tolerance by upregulating the expression of *hsf-1* and *HSPs*. (**A**) Survival curves of N2 cultured at 37 °C; *p* was calculated using the log-rank test. *n* ≥ 60. (**B**) Effects of DnAE on the mRNA expression of *hsf-1* and its target genes in N2 treated with 1 mg/mL DnAE for 96 h (*n* ≥ 1000, * *p* < 0.05, ** *p* < 0.01, **** *p* < 0.0001, multiple *t* tests). (**C**) Quantification of *hsp-16.2* expression in the *hsp-16.2p*::GFP mutant strain TJ375 (*n* ≥ 20, * *p* < 0.05, ** *p* < 0.01, **** *p* < 0.0001, *t* test). (**D**) Survival curve of the *hsf-1* mutant strain *hsf-1(sy441)* treated with or without 1 mg/mL DnAE at 20 °C (*n* ≥ 60, *p* > 0.05, log-rank test).

Due to the close relationship between lifespan extension and stress resistance [24], we further investigated whether DnAE could extend the lifespan of *C. elegans* under thermal stress. In the thermal stress experiment, wild-type worms were cultured for 5 days and then exposed to 37 °C. Their survival and mortality rates were recorded hourly. The results showed that, compared to the control group, DnAE increased the survival rate of *C. elegans* under heat stress and extended their lifespan by 34.09% (*p* = 0.007) (Figure 3A and Table S7). HSF-1 is the main transcription factor that affects the heat resistance of *C. elegans*, and downstream target proteins such as HSP-16.2, HSP-6, and HSP-60 are involved in the response to stress. In the present study, DnAE significantly increased the mRNA levels of *hsf-1*- and *hsp*-related genes (Figure 3B and Table S14). To confirm the link between HSF-1 and DnAE, we quantified *hsp-16.2* expression using the *hsp-16.2p*::green fluorescent protein (GFP) mutant strain TJ375. Our results showed that DnAE enhanced the fluorescence level of TJ375 cells (Figure 3C and Table S10), while it lost the ability to extend the nematode lifespan in the *hsf-1* ineffective mutant *hsf-1(sy441* and Table S11*)* (Figure 3D).

### 2.4. DnAE Enhances Antioxidant Activity in C. elegans

**Figure 4:**
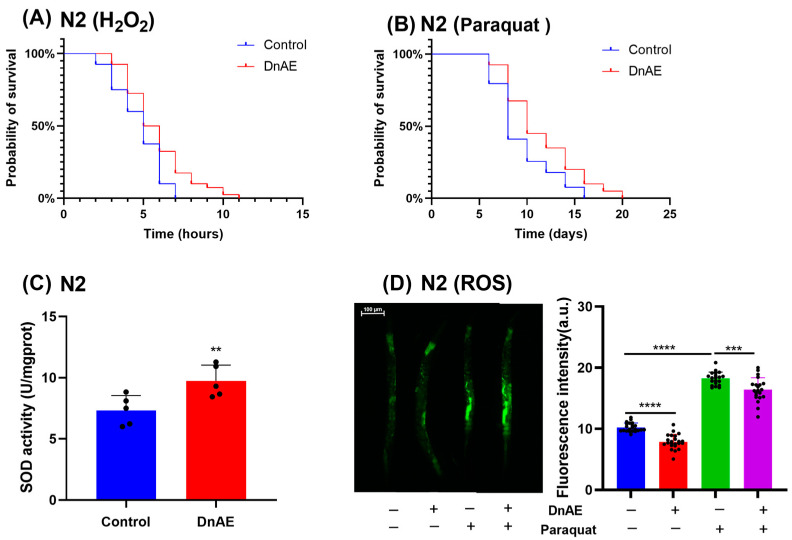
Effect of DnAE on the antioxidant capacity of N2. Survival curve of N2 treated with or without 1 mg/mL DnAE under oxidative stress induced by (**A**) 10 mM H_2_O_2_ and (**B**) 10 mM paraquat; *p* was calculated using the log-rank test. *n* ≥ 60. (**C**) SOD activity in *C. elegans*. L4-stage N2 was cultured for 72 h and treated with or without 1 mg/mL DnAE (*n* ≥ 1000, ** *p* < 0.01, *t* test). (**D**) Representative fluorescence images showing ROS in N2 treated with DnAE and 4 mM paraquat. The bar graph indicates the fluorescence intensity of N2 (*n* ≥ 20, *** *p* < 0.001, **** *p* < 0.0001, *t* test).

Oxidative damage is a key factor in aging and manifests as the level of free radicals in the body [35]. Consequently, drugs with antioxidant capacity are often considered almost analogous to antiaging drugs. To investigate the effect of DnAE on the antioxidant capacity of *C. elegans*, we exposed the nematodes to hydrogen peroxide- and paraquat-induced oxidative environments and measured their survival time. The DnAE-treated groups exhibited excellent antioxidant capacity, with average lifespan extensions of 23.16% and 21.87% under acute and chronic oxidative stress, respectively, compared to those in the control group (*p* < 0.001) (Figure 4A,B and Table S7). Additionally, we measured the following molecular markers of antioxidant defense: SOD and ROS. ROS are oxidative stress substances produced within cells, and SOD can help clear ROS and reduce the degree of oxidative stress reactions. DnAE prolonged the lifetime of N2 under oxidative stress, reduced ROS levels, and increased SOD activity (Figure 4C,D and Tables S8 and S9).

### 2.5. DnAE Promotes the Nuclear Localization of DAF-16 and the Expression of SOD-3 and GST-4

**Figure 5:**
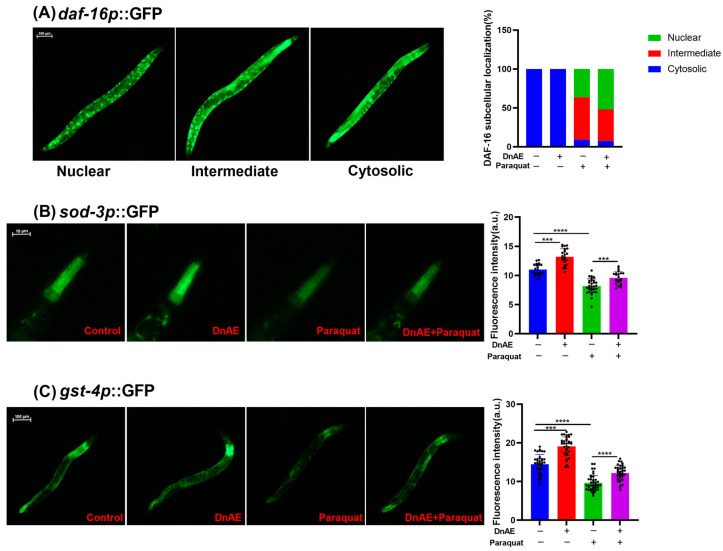
Effects of DnAE on DAF-16 cellular translocation and SOD-3 and GST-4 expression in GFP-tagged N2. (**A**) Percentage of DAF-16 localization determined by quantitative analysis of the cytosolic, intermediate, and nuclear localization of DAF-16 in *daf-16p::daf-16a/b*::GFP worms. *n* ≥ 20. (**B**) Fluorescence intensity of *sod-3p*::GFP in CF1553 strain with or without 1 mg/mL DnAE (*n* ≥ 20, *** *p* < 0.001,**** *p* < 0.0001, *t* test). (**C**) Fluorescence intensity of *gst-4p*::GFP in CL2166 worms with or without 1 mg/mL DnAE. *n* ≥ 20.

To gain a deeper understanding of the molecular mechanism by which DnAE extends the lifespan of wild-type *C. elegans* in view of the beneficial effects of DnAE on heat and oxidative stress, we tested whether DnAE could affect the transcriptional activity of the previously studied longevity-associated transcription factor DAF-16. DAF-16 is the ortholog of the forkhead box O (FOXO) family in humans [36]. The activation of DAF-16 can lead to the increased expression of antioxidant enzymes, such as SOD, which helps protect cells against oxidative damage and contributes to lifespan extension. We used the TJ356 *daf-16p::daf-16a/b*::GFP mutant to study the subcellular localization of DAF-16 and the expression of its target genes. As shown in Figure 5A, DnAE increased the number of worms with nuclear localization of DAF-16 after treatment with paraquat. As SOD-3 and GST-4 are essential downstream target proteins of the DAF-16 transcription factor, their expression was visualized by exploiting the CF1553 and CL2166 mutant strains (Figure 5B,C). Compared to that in the control group, the fluorescence intensity in the DnAE treatment group was significantly enhanced, implying increased SOD-3 and GST-4 expression. Paraquat substantially depletes antioxidant enzymes in *C. elegans*. DnAE increased the fluorescence intensity of SOD-3 and GST-4 after treatment with 4 mM paraquat, enhancing *C. elegans* resistance (Table S10).

### 2.6. DnAE-Mediated Lifespan Extension in C. elegans Is Dependent on DAF-16

**Figure 6:**
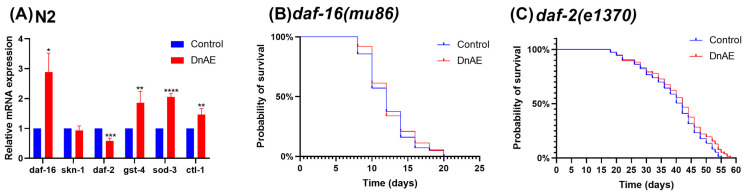
DnAE-mediated extension of the *C. elegans* lifespan via DAF-16. (**A**) mRNA expression of *skn-1*, *daf-16*, and related target genes in N2 treated with DnAE for 96 h (*n* ≥ 1000, * *p* < 0.05, ** *p* < 0.01, *** *p* < 0.001, **** *p* < 0.0001, multiple *t* tests). (**B**) Survival curves of DnAE-treated or untreated *daf-16(mu86)* mutant strain at 20 °C (*n* ≥ 60, *p* > 0.05, log-rank test). (**C**) Survival curves of DnAE-treated or untreated *daf-2(e1370)* mutant strain at 20 °C (*n* ≥ 60, *p* > 0.05, log-rank test).

Apart from DAF-16, the transcription factor SKN-1 is another well-known longevity factor that plays an important role in the oxidative stress response. To clarify which signaling pathway is regulated by DnAE to prolong the lifespan of *C. elegans*, we examined the expression of *skn-1*, *daf-16*, and related target genes in N2 under DnAE treatment. Our findings demonstrate that while *skn-1* expression remained unaffected, DnAE significantly upregulated the expression of *daf-16* and its downstream genes such as *sod-3*, *gst-4*, and *ctl-1* and decreased the expression of its upstream gene *daf-2* (Figure 6A and Table S14). Furthermore, after DnAE administration, we found that DnAE had no effect on the lifespan of *daf-16 (mu86)* or *daf-2 (e1370)* mutant strains (Figure 6B,C and Table S11). *daf-2* encodes the sole *C. elegans* homolog of the insulin/IGF-1 receptor, and its degradation stimulates DAF-16 activity, promoting longevity [37]. Therefore, the extension of the lifespan of *C. elegans* by DnAE mainly depends on DAF-16 from the IIS pathway.

### 2.7. DnAE Decreases the Progression of Aging-Related Diseases

**Figure 7:**
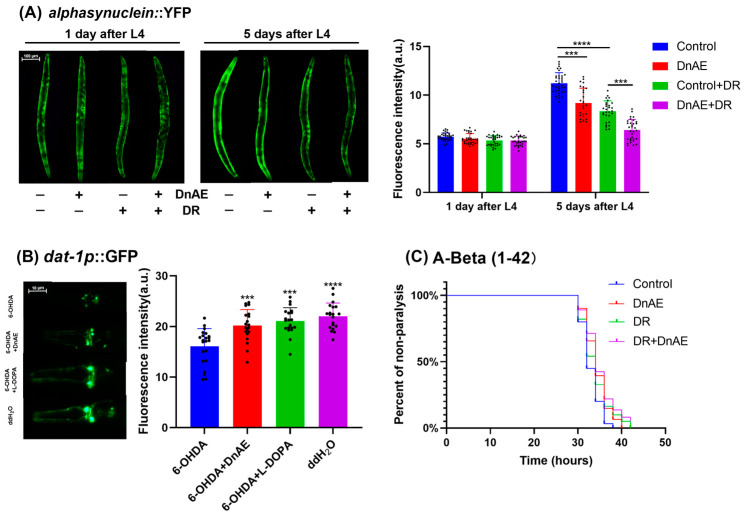
Effect of DnAE on aging-related diseases in mutant strains. (**A**) Representative fluorescence images showing α-synuclein aggregation in the NL5901 (*unc-54p::alpha synuclein*::YFP) strain treated with DnAE. The fluorescence intensity of α-synuclein is presented by the bar graph (*n* ≥ 20, *** *p* ≤ 0.001, **** *p* < 0.0001, *t* test). (**B**) Representative fluorescence images showing dopaminergic neurons with GFP signals in DnAE-treated bz555 (*dat-1p*::GFP) strain. The bar graph indicates the fluorescence intensity (*n* ≥ 20, *** *p* ≤ 0.001, **** *p* < 0.0001, *t* test). (**C**) The paralysis curve of the CL4176 (*myo-3p*::A-Beta (1-42)::let-851 3′UTR) mutant strain treated with or without 1 mg/mL DnAE at 25 °C. *n* ≥ 20.

PD is characterized by the accumulation of α-synuclein in the substantia nigra and the degeneration of dopaminergic neurons [38]. *C. elegans* lacks a homolog of α-synuclein, so a transgenic strain expressing human α-synuclein in body wall muscle cells was created to study the pathogenicity of α-synuclein [39]. The transgenic strain NL5901 expresses α-synuclein fused with yellow fluorescent protein (YFP). Dietary restriction (DR) significantly inhibits protein toxicity and age-related paralysis in the *C. elegans* model through a mechanism distinct from the IIS pathway [40]. After treatment with 1 mg/mL DnAE, we observed no noticeable change in α-synuclein aggregation on the first day, but there was a significant reduction after five days. Additionally, DnAE treatment notably enhanced the inhibition of α-synuclein aggregation compared to that in the positive control group under DR conditions (Figure 7A and Table S12).

Dopaminergic neuron degeneration is readily induced by neurotoxins such as 6-hydroxydopamine (6-OHDA) [41]. Therefore, another transgenic strain, BZ555, expressing GFP in dopaminergic neurons, was used to study dopaminergic neuron degeneration. After treating *C. elegans* with 6-OHDA for 72 h, fluorescence images of its head neurons were taken. The results demonstrated that 6-OHDA treatment reduced the average fluorescence intensity of BZ555 in the absence of DnAE, while the addition of DnAE improved this effect (Figure 7B). These results revealed DnAE-delayed dopaminergic neuron degeneration in BZ555. Its protective effect was equivalent to that of the anti-PD drug levodopa (Figure 7B and Table S12).

β-Amyloid is the main cause of neuronal degeneration and death around senile plaques in the brains of AD patients. High temperatures can induce its expression in the muscle of the transgenic strain CL4176, resulting in paralysis of the strain [42]. DnAE delayed the paralysis of the CL4176 strain and reduced the toxicity of the β-amyloid protein, In the DR model, CL4176 paralysis was delayed by 7.01%, which increased to 10.01% in the presence of DnAE (Figure 7C and Table S13).

## 3. Discussion

Various components of DnEA have significant pharmacological effects. Dendrobine, known for its antiaging and neuroprotective effects, inhibits dopamine-related neuronal apoptosis in PD models [43] and reduces SASP factor expression in chondrocytes, suggesting potential for osteoarthritis treatment [44]. Dendroxine protects PC12 cells from Aβ1-42-induced neurotoxicity by inhibiting CDK5 [45]. Nobilonine reduces lipopolysaccharides in rats [46]. With a wide range of physiological functions, oleic acid reduces the risk of cardiovascular disease, autoimmune disorders, and cancer and aids wound healing [47]. Therefore, DnAE has notable pharmacological effects on aging and aging-related diseases.

Contrary to earlier findings [30,48], DnAE increased *C. elegans*’ longevity and slowed the buildup of lipofuscin, but it had no effect on their bending frequency, pharyngeal pumping rate, or ability to lay eggs over a given amount of time. Thus, we suggest that DnAE could function via a different mechanism than calorie restriction, increased exercise, or the suppression of reproduction.

There is a strong correlation between the extension of the lifespan of *C. elegans* and their ability to resist stress, including heat stress. DnAE significantly increased the survival time of *C. elegans* exposed to high temperatures, enhancing heat resistance. Additionally, DnAE upregulated the expression of *hsf-1* and HSPs. HSF-1 is a transcription factor that is pivotal in the heat stress response. When cells experience adverse environmental conditions such as high temperatures and oxidative stress, HSF-1 can activate the expression of a series of HSPs that protect cells from stress-induced damage. HSPs act as molecular chaperones and regulate protein folding, thereby safeguarding other proteins against stress-induced damage [40]. Some HSPs have been shown to inhibit virus proliferation by interacting with viruses and activating the immune pathway to protect host cells during viral infections, indicating antiviral properties [49]. These findings are consistent with the reported antiviral effects of *D. nobile* [50]. Therefore, DnAE delayed the aging process of *C. elegans* by increasing its tolerance to adversity by upregulating the expression of *hsf-1* and HSPs.

*D. nobile* contains several chemical components that exert antioxidant effects [51]. Consistent with this finding, DnAE significantly extended the survival time of *C. elegans* exposed to H_2_O_2_ and paraquat. We believe that the antioxidant properties of DnAE are crucial for delaying the aging process.

SKN-1, a regulatory factor for *C. elegans*’ responses to environmental stress, is a homologous gene to Nrf2 [52,53]. Despite some evidence that *D. nobile* activates the Nrf2 pathway to exert its biological activity [11,54,55], we discovered that DnAE did not increase *skn-1* expression. Daf-16 activation and Daf-2 degradation are the primary regulators of the IIS pathway that prolong the lifespan of C. *elegans* [56]. According to our findings, DnAE promoted DAF-16 entry into the nucleus through the cytoplasm and enhanced the expression of *daf-16* and its downstream regulatory genes *sod-3*, *ctl-1*, and *gst-4*. Moreover, DnAE downregulated the expression of *daf-2*, which stimulates DAF-16 activity but did not extend the lifespan of the *daf-16(mu86)* or *daf-2(e1370)* mutant strains. These findings suggest that DnAE may prolong the lifespan of *C. elegans* by inhibiting the IIS pathway.

Neurodegenerative diseases caused by aging have always been a challenge in medicine. Water extracts of *D. nobile* can inhibit neuronal apoptosis, enhance the expression of nerve growth factors, and provide neuroprotection for rats with hypoxic-ischemic brain injury [57]. Since nematodes lack homologous genes for PD/AD, we used transgenic nematode strains and found that DnAE protected DA neurons from 6-OHDA-induced neurodegeneration, reduced the aggregation of α-synuclein, and lowered Aβ-induced neurotoxicity. This finding is consistent with the neuroprotective effect of *D. nobile* extract in other animal models [58]. According to previous reports, DR inhibits protein toxicity and age-related paralysis in *C. elegans* models through pathways unrelated to daf-16 [40]. In the DR model, DnAE strengthened the inhibition of α-synuclein aggregation and delayed the paralysis induced by Aβ, indicating that DnAE exerts neuroprotective effects through pathways different from those in DR. In addition, research has shown a close relationship between DAF-16 and the nervous system. For example, gastrodin reduces α-synuclein accumulation in nematodes through the DAF-2/DAF-16 signaling pathway [59], and cannabidivarin prevents DA-related neuronal degeneration through DAF-16 [60]. The activation of DAF-16 improves the royal jelly protein balance and reduces β-amyloid toxicity [61]. Therefore, we speculated that *D. nobile* extract provides neuroprotection by promoting DAF-16 expression, but extensive experimental data are required to confirm this.

## 4. Conclusions

**Figure 8:**
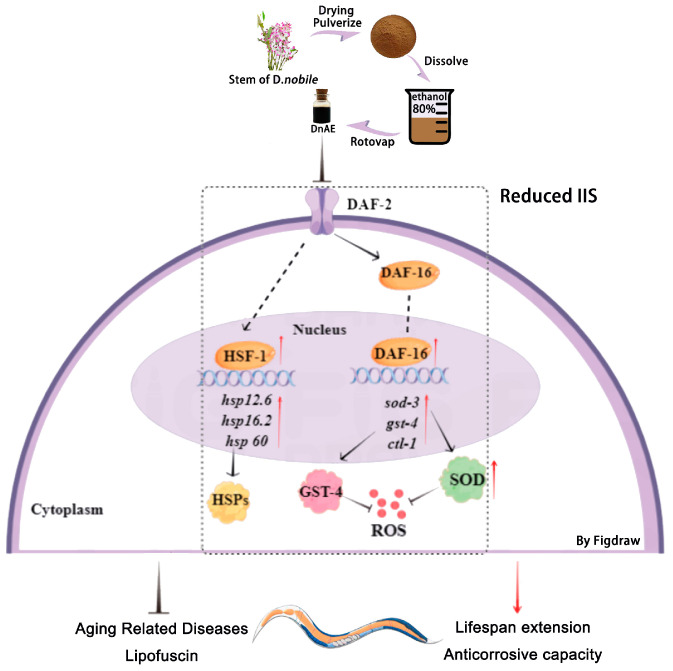
DnAE increases stress tolerance, delays the progression of aging-related diseases, and extends the lifespan of *C. elegans* via DAF-16 and HSF-1. Red arrow: stimulatory effect, identified by our experiments; black arrow: stimulatory effect; dash line arrow: speculated stimulatory effect; dash line: translocation process; solid “T” line: inhibitory effect.

DnAE slowed the accumulation of lipofuscin, reduced the ROS content, and increased the SOD activity of N2. Moreover, it prolonged the lifespan of N2 under oxidative stress and heat stress, which was achieved by upregulating the expression of *daf-16* and *hsf-1*, promoting the nuclear localization of DAF-16, and increasing the expression of *sod-3*, *gst-4*, and *ctl-1*. Additionally, DnAE alleviated 6-OHDA-induced DA-related neuronal degeneration and reduced Aβ-induced toxicity. These results highlight the potential of DnAE for further investigation in extending lifespan and treating age-related diseases (Figure 8).

## 5. Materials and Methods

### 5.1. Strains and DnAE Preparation

The following strains were obtained from the Caenorhabditis Genetics Center (CGC) and maintained at the appropriate temperature as per previous reports [62] unless otherwise specified: N2 (Bristol, wild-type) [63], CL2166 dvIs19 [(pAF15) *gst-4p*::GFP::NLS] [64], TJ356 zIs356 [*daf-16p::daf-16a/b*::GFP + rol-6(su1006)] [21], NL5901 pkIs2386 [*unc-54p::alphasynuclein*::YFP + *unc-119*(+)] [39], CF1038 *daf-16 (mu86)* [65], BZ555 egIs1 (*dat-1p*::GFP) [66], CF1553 muIs84 [(pAD76) *sod-3p*::GFP + *rol-6(su1006)*] [67], TJ375 gpIs1 [*hsp-16.2p*::GFP] [68], PS3551 *hsf-1(sy441)* [68], CL4176 dvIs27 [*myo-3p*::A-Beta (1-42)::let-851 3′UTR) + *rol-6(su1006)*] [42], CB1370 *daf-2(e1370)* [65]. The strains were cultured at 20 °C on NGM plates containing OP50 unless otherwise specified. *D. nobile* was purchased from Sichuan Gentle Orchid Agricultural Science and Technology Co., Ltd. (Ziyang, China). It was dried in an oven at 60 °C and pulverized. The resulting powder was sifted through an 80-mesh sieve, mixed with petroleum ether, and subjected to ultrasonic oscillation for 30 min to produce a solid–liquid mixture. The mixture was subsequently filtered, and the remaining residues were ultrasonicated with 80%, 75%, and 70% ethyl alcohol (1:50 g/mL *w*/*v*) at 50 °C for 30 min before refiltering. Finally, all alcohol extracts were combined and evaporated to obtain DnAE.

### 5.2. UHPLC-Q-TOF-MS/MS Compound Identification

Compound identification of DnAE was carried out using a UHPLC Agilent 12900 series instrument (Agilent Technologies, Santa Clara, CA, USA) and an ACQUITY UPLC BEH C-18 (Waters Corp, Milford, MA, USA) analytical column (100 mm × 2.1 mm). An AB Triple TOF 6600 (SCIEX, Framingham, MA, USA) mass spectrometer was used to collect the primary and secondary spectra of the compounds. The chromatographic and mass spectrometry conditions followed previous methods [69]. The structures of the compounds were identified by comparing their molecular weights (molecular mass errors less than 10 ppm), secondary fragmentation determination spectra, and retention times in the Shanghai Applied Protein Technology database and public databases Mass Bank, Metlin, HMDB, and MoNA. These results were subsequently checked and confirmed by comparison with the standards and literature.

### 5.3. Lifespan Assay

DnAE was diluted with water to concentrations of 200 μg/mL, 500 μg/mL, and 1 mg/mL after being dissolved in ethanol to a concentration of 80 mg/mL. DnAE was applied to the prepared NGM plates at various concentrations and allowed to blow dry, after which, 200 μL OP50 was added to the NGM plates. At least 60 synchronized L4-stage *C. elegans* were selected and placed on NGM plates for the treatment and control groups. The time of synchronization was recorded as day 0. The worms were cultured at 20 °C, transferred to new NGM plates with or without drugs every 24 h, and counted for survival, death, or missing numbers. To accurately capture the number of deaths during natural growth, dead worms were counted beginning on day 8. Worms that did not respond when lightly touched on the head with a worm pick were considered dead. The experiment was repeated three times, as shown in Table S1.

### 5.4. Reproduction Assay

Three synchronized L4-stage *C. elegans* were selected and equally divided into treatment and control groups. The worms were transferred to NGM plates containing 1 mg/mL DnAE for the treatment group or solvent without DnAE for the control group. The worms were transferred to new NGM plates every 24 h, and the number of offspring produced on the previous plates was recorded. Self-fertilization of *C. elegans* was observed for 5 days after the L4 stage to calculate the average and total number of eggs produced per hermaphroditic worm. The experiment was repeated three times and the numbers of *C. elegans* per treatment are shown in Table S.

### 5.5. Bending Assay

This study aimed to assess the effect of *D. nobile* on *C. elegans*’ motility. At least 20 synchronized L4-stage worms were transferred to NGM plates containing 1 mg/mL DnAE or DnAE-free solvent for the treatment or control groups, respectively. The worms were transferred to new NGM plates every 48 h and cultured at 20 °C for 72 h before the number of body bends was recorded for 20 s. These measurements were used to assess motility. The experiment was repeated three times, as shown in Table S5.

### 5.6. Pharyngeal Pumping Assay

At least 20 synchronized L4-stage *C. elegans* were transferred to NGM plates and treated with 1 mg/mL DnAE or blank solvent for the treatment or control groups, respectively. The treatment group was further divided into subgroups according to DnAE concentrations. The worms were transferred to new NGM plates every 48 h and cultured at 20 °C for 72 h. Then, the worms were observed using a microscope, and the number of pharyngeal pumping events during a 10 s period was recorded. The experiment was repeated three times, as shown in Table S4.

### 5.7. Oxidative Stress Resistance Assay

The dosing and control groups were treated with or without 1 mg/mL DnAE. At least 60 synchronized L4-stage nematodes were selected and transferred to NGM plates for the treatment and control groups. After 48 h, the NGM plates were changed. Into new plates, 400 μL of 10 mM H_2_O_2_ was introduced after 72 h; this time point was then taken as 0 h and the number of dead *C. elegans* was noted every hour to calculate the survival rate using criteria similar to the lifespan assay. The experiment was repeated three times, as shown in Table S7.

### 5.8. Chronic Oxidative Stress Resistance Assay

At least 60 synchronized L4-stage *C. elegans* were selected and transferred to DnAE-containing or DnAE-free NGM culture dishes. Then, 200 μL of 10 mM glyphosate solution was added to each culture dish. The *C. elegans* were relocated to new plates and counted for dead and surviving numbers every 48 h until all the worms were dead. The assay was carried out in three parallel groups. The experiment was repeated three times, as shown in Table S7.

### 5.9. Thermal Stress Resistance Assay

The dosing and control groups were treated with or without 1 mg/mL DnAE. After transferring at least 40 synchronized L4-stage nematodes into NGM dishes, they were treated with either 1 mg/mL DnAE or solvent only and cultivated for 5 days at 20 °C. Then, the temperature was raised to 37 °C and the number of dead worms was counted every hour. When the worms no longer responded to being touched by the platinum wire, they were considered dead. The experiment was repeated three times, as shown in Table S7.

### 5.10. Determination of Lipofuscin

Wild-type nematodes were cultured according to the lifespan assay. After 5 and 10 days of culture, the fluorescence intensity was observed using an inverted fluorescence microscope (NIKON Eclipse Ts2R, Nikon, Tokyo, Japan) under conditions of excitation at 340 nm and emission at 430 nm. The average fluorescence intensity of the entire body of *C. elegans* was then analyzed using ImageJ v1.53. The experiment was repeated three times and at least 20 nematodes were included in each group, as shown in Table S6.

### 5.11. Determination of ROS Levels

ROS levels were quantified using the cell membrane-permeable reactive oxygen species (ROS) detection probe H2DCFH-DA. After synchronization of the wild-type nematodes, L4-stage nematodes were selected and transferred to new NGM dishes with or without 1 mg/mL DnAE every 48 h. In the positive control group, an additional 4 mM paraquat was added. After 5 days of incubation, the nematodes were transferred to an M9 buffer containing 100 μM H2DCFH-DA and incubated for 2 h at 20 °C in a light-protected incubator. The fluorescence intensity was observed with an inverted fluorescence microscope (Nikon eclipse Ts2R) at an excitation wavelength of 488 nm, and the average fluorescence intensity of the entire body was subsequently analyzed using ImageJ. At least 20 *C. elegans* were used per group and the experiment was repeated three times, as shown in Table S8.

### 5.12. Determination of SOD Activity

This experiment involved a treatment group and a control group treated with or without 1 mg/mL DnAE. After synchronization at the L4 stage, approximately 1000 nematodes were transferred to each group every 48 h. After 5 days of normal incubation, the nematode lysate was prepared using an ultrasonic cell disrupter under the following conditions: ultrasonic power of 200 W, cycle time of 20 s, interval of 10 s, and 10 cycles. The total protein concentrations of the lysate were quantified using the BCA protein assay kit (Beyotime, P0012). SOD activity was detected using a commercial kit (Nanjing Jiancheng Bioengineering Institute, A001-3), mostly following Chen Xu’s protocol [70]. Briefly, the lysate was added to a 96-well plate supplemented with superoxide anion radical and 2-(4-iodophenyl)-3-(4-nitrophenyl)-5-(2,4-disulfophenyl)-2H-tetrazolium sodium salt (WST-1) and incubated at 37 °C for 20 min to produce SWT-1 formazan. The absorbance of the formazan was measured at 450 nm under a microtiter plate reader (Thermo, Varioskan Flash, Waltham, MA, USA), which was used for calculating the activity of SOD following the manufacturer’s instructions. The experiment was repeated three times, as shown in Table S9.

### 5.13. Quantitative Analysis of Fluorescence Intensity

Through fluorescence intensity quantification analysis, we used the mutant strains BZ555, NL5901, CL2166, CF1553, and TJ375 to analyze whether DnAE affects dopamine-related neuronal loss, the aggregation of α-synuclein, and the expression of GST-4, SOD-3, and HSP-16.2. Each plate contained 50 μM FUDR to inhibit egg hatching. The BZ555 mutant strain was subjected to a one-hour immersion in a 50 mM solution of 6-hydroxydopamine (6-OHDA) to serve as the model group for dopamine neuron loss. In contrast, the positive control group was treated with 2 mM levodopa (L-DOPA). The TJ356 mutant strain was subjected to a one-hour treatment at 35 °C before imaging as a stress model. The establishment of the CL2166 and CF1553 model groups followed the same strategy described in Section 5.11. The DR model was established with the NL5901 strain, consistent with previous reports [40]. Adult worms were transferred to NGM plates without OP50 every two days and cultured for five days. Approximately 60 late L4 larvae or young adults (mutants) were transferred to plates containing 1 mg/mL DnAE or etOH and cultured with inactivated OP50 at 20 °C until day 5. An inverted fluorescence microscope (Nikon eclipse Ts2R) was used to take photographs. ImageJ was used to analyze the average fluorescence intensity in the heads of the BZ555 and CF1553 mutants, while the overall average fluorescence intensity was analyzed for the remaining mutant strains. Real-time images were obtained from at least 20 worms per group and the experiment was repeated three times, as shown in Tables S10 and S12.

### 5.14. Nuclear Localization of DAF-16

As described for the lifespan assay, the TJ356 strain was cultured at 20 °C for four days. Subsequently, it was treated with freshly prepared 4 mM paraquat for 24 h [71]. The nuclear translocation of DAF-16 was observed under a fluorescence inverted microscope and images were taken. The obtained data, which were labeled uniformly, were presented to a third party. The data were categorized into three types: nuclear entry, intermediate state, and cytoplasmic entry. The quantity of each category was counted and the corresponding proportions were calculated. The experiment was repeated three times and the number of *C. elegans* per treatment was not less than 20, as shown in Table S10.

### 5.15. Paralysis Assay

The CL4176 worms were incubated at 15 °C until the L3 stage, transferred to NGM plates containing 1 mg/mL DnAE, and incubated at 25 °C, according to the methods of Drake, Jennifer [42]. Paralyzed nematodes were counted every 2 h. The DR model group underwent the same treatment as described in Section 5.13 for NL5901. This experiment was independently repeated three times with a minimum of 40 worms in each group, as shown in Table S13.

### 5.16. Quantitative Real-Time PCR Assay

Approximately 2000 synchronized young adult nematodes were transferred to DnAE-containing or DnAE-free NGM plates and then maintained at 20 °C for 24 h. Total RNA was extracted using RNAiso Plus (Takara, Kyoto, Japan) and converted into cDNA with a High Capacity cDNA Reverse Transcription Kit (Applied Biosystems, Waltham, MA, USA). Quantitative real-time PCR was carried out using Power SYBR Green PCR Master Mix (Applied Biosystems, Waltham, MA, USA) in a QuantStudio 6 Flex system. The expression levels of the genes were calculated using the 2^−ΔΔCT^ method and normalized to the expression of the cdc-42 gene. The statistical significance of the differences was determined using the *t* test. The experiment was repeated three times, as shown in Tables S14 and S15.

### 5.17. Statistical Analysis

All the experiments were performed in triplicate. The data are presented as the mean ± SEM unless specifically indicated otherwise. All the statistical analyses included *t* tests or log-rank tests. All the figures were generated using GraphPad Prism 9, SPSS 26.

## Figures and Tables

**Table 1:** Composition characteristics of DnAE.

Number	Compounds	Adduct	Formula	*m*/*z*	Retention Time (Min)	Peak Area	Reference
1	Palmitic amide **	[M+H]^+^	C_16_H_33_NO	256.2637	10.865	75,870,888	
2	C2 **	[M−H_2_O+H]^+^	C_44_H_86_NO_7_P	284.2942	11.453	74,055,912	
3	Glyceryl monostearate **	[M+H]^+^	C_21_H_42_O_4_	359.3146	11.586	19,812,174	
4	Linoleic acid *	[M+H−H_2_O]^+^	C_18_H_32_O_2_	263.2356	10.686	11,779,023	[25]
5	2-Palmitoylglycerol *	[M+H]^+^	C_19_H_38_O_4_	331.2829	11.069	4,608,534	
6	C6 **	[M+Na]^+^	C_24_H_30_O_6_	437.1923	7.965	3,485,238	
7	Triethanolamine **	[M+H]^+^	C_6_H_15_NO_3_	150.1106	11.376	2,495,882	
8	Palmitic acid *	[M-H]^−^	C_16_H_32_O_2_	255.2328	11.324	2,262,816	
9	C9 **	[M+ACN+H]^+^	C_22_H_30_O_6_	432.2376	7.965	1,424,730	
10	Mubironine B **	[M+H]^+^	C_15_H_23_NO_2_	250.1789	7.038	1,252,497	[26]
11	Quinic acid *	[M−H]^−^	C_7_H_12_O_6_	191.0573	0.617	897,605	[27]
12	2-Aminooctadecane-1,3,4-triol *	[M+H]^+^	C_18_H_39_NO_3_	318.2985	8.293	759,759	
13	C13 **	[M+H]^+^	C_22_H_39_NO	334.3099	11.38	707,127	
14	Dendroside G **	[M+Na]^+^	C_21_H_34_O_10_	469.2029	4.291	626,858	[28]
15	Sucrose **	[M−H]^−^	C_12_H_22_O_11_	341.1098	0.659	613,066	
16	C16 **	[M+H]^+^	C_21_H_33_N_3_O_3_	376.2586	7.091	608,355	
17	Glyceryl palmitate **	[M+NH_4_]^+^	C_19_H_38_O_4_	348.307	11.069	439,586	
18	Dendroxine **	[M+H]^+^	C_17_H_25_NO_3_	292.1917	7.07	436,709	[29]
19	C19 **	[M+Na]^+^	C_21_H_32_O_9_	451.1933	5.221	397,304	
20	Dendrobine *	[M+H]^+^	C_16_H_25_NO_2_	264.1934	10.865	376,165	[30]
21	13′-Hydroxy-α-tocopherol	[M+H−H_2_O]^+^	C_29_H_50_O_3_	429.3703	11.453	230,873	
22	Oleic acid *	[M-H]^−^	C_18_H_34_O_2_	281.2469	11.586	188,108	
23	Nobilonine **	[M+H]^+^	C_17_H_27_NO_3_	294.2039	4.174	157,383	[26]
24	Dibutyl phthalate **	[M+H]^+^	C_16_H_22_O_4_	279.1582	8.906	127,790	[31]
25	Dendroside F **	[M+Na]^+^	C_21_H_34_O_9_	453.20569	2.746	124,435	[28]
26	Gamma-Linolenic acid *	[M−H]^−^	C_18_H_30_O_2_	277.2161	10.686	32,712	
27	Dendronobilin F **	[M−H]^−^	C_15_H_22_O_5_	281.1403	8.168	7220	[30]

* Confirmed with standards. ** Tentatively identified based on library and/or literature data. C2: [3-[(1Z,9Z)-Octadeca-1,9-dienoxy]-2-octadecanoyloxypropyl]2-(trimethylazaniumyl)ethyl phosphate. C6: Methyl3-[(1E,3E)-3,5-dimethyl-1,3-heptadien-1-yl]-8-hydroxy-6a,8-dimethyl-6-oxo-6a,8,9,9a-tetrahydro-6H-furo [2,3-h] isochromene-9-carboxylate. C9: [1,14-dihydroxy-8-(hydroxymethyl)-4,12,12,15-tetramethyl-5-oxo-13-tetracyclo [8.5.0.02,6.011,13] pentadeca-3,8-dienyl] acetate. C13: 2,4,12-octadecatrienoic acid isobutylamide. C16: 7″-ethyl-6-methyl-4″,7″-dihydro-3″H-dispiro[oxane-2,6′-[7,9,12] triazatricyclo[6.3.1.04,12]dodecane-10′,2″-oxepin]-8′-en-3-ol. C19: 4-Hydroxy-6-methyl-3-methylidene-5-[5-[(2R,3R,4S,5S,6R)-3,4,5-trihydroxy-6-(hydroxymethyl)oxan-2-yl]oxypentan-2-yl]-3a,4,7,7a-tetrahydro-1-benzofuran-2-one.

## Data Availability

Data are contained within the article.

## Supplementary Materials

The following supporting information can be downloaded at https://www.mdpi.com/article/10.3390/molecules29040908/s1: Figure S1: Mass spectrum of the compounds contained in DnAE; Table S1: Lifespan of wild-type nematode (N2) treated with DnAE at different concentrations; Table S2: Lifespan of wild-type nematode (N2) treated with 214 μmol/mL EtOH (consistent with the concentration of alcohol contained in 1 mg/mL DnAE); Table S3: Effect of DnAE on oviposition rate of N2 worms; Table S4: Effect of DnAE on pharyngeal pumping in wild-type nematodes (N2); Table S5: Effect of DnAE on body movement of wild-type nematodes (N2); Table S6: Effect of DnAE on lipofuscin in wild-type nematodes (N2); Table S7: Effect of DnAE on resistance to high temperatures and oxidation of wild-type nematodes (N2); Table S8: Effect of DnAE on ROS levels of N2 nematodes; Table S9: Effect of DnAE on SOD levels of N2 nematodes; Table S10: Effect of DnAE on expression of HSP-12.6, SOD-3, GST-4, and DAF-16 in mutant nematodes; Table S11: Effect of DnAE on lifespan of mutant nematodes; Table S12: Effects of DnAE on Parkinson’s disease model; Table S13: Effects of DnAE on Alzheimer’s disease model; Table S14: Effect of DnAE on gene expression at mRNA level in nematodes; Table S15: Primers used for the analysis of mRNA expression levels in nematodes.
