# Supplementary material for: Dendrobium Nobile Alcohol Extract Extends the Lifespan of Caenorhabditis elegans via hsf-1 and daf-16
Source: Molecules. 2024 Feb 19;29(4):908. doi: 10.3390/molecules29040908 (PMC10891841; doi:10.3390/molecules29040908)
Supplement: Supplementary file 1 [file molecules-29-00908-s001.zip › molecules_cmpd-check-list.pdf]

molecules

MDPI

Chemical Compounds Characterization Checklist

Manuscript ID: molecules-2442023

Submitting Author: Runlan Wan

Compound			Characterization											
Compound Nr.	New	Known (Ref. in main text)	Physical State (purity %)	1H NMR	13C NMR	2D NMR	HRMS	IR	XRD	GC/ HPLC				Data in SI
1	☐	itic amide	37.23	☐	☐	☐	☑	☐	☐	☑	☐	☐	☐	☐
2	☐	phosphate	36.34	☐	☐	☐	☑	☐	☐	☑	☐	☐	☐	☐
3	☐	ostearate	9.72	☐	☐	☐	☑	☐	☐	☑	☐	☐	☐	☐
4	☐	leic acid	5.78	☐	☐	☐	☑	☐	☐	☑	☐	☐	☐	☐
5	☐	lglycerol	2.26	☐	☐	☐	☑	☐	☐	☑	☐	☐	☐	☐
6	☐	rboxylate	1.71	☐	☐	☐	☑	☐	☐	☑	☐	☐	☐	☐
7	☐	anolamine	1.22	☐	☐	☐	☑	☐	☐	☑	☐	☐	☐	☐
8	☐	itic acid	1.11	☐	☐	☐	☑	☐	☐	☑	☐	☐	☐	☐
9	☐	l] acetate	0.69	☐	☐	☐	☑	☐	☐	☑	☐	☐	☐	☐
10	☐	ironine B	0.61	☐	☐	☐	☑	☐	☐	☑	☐	☐	☐	☐
11	☐	inic acid	0.44	☐	☐	☐	☑	☐	☐	☑	☐	☐	☐	☐
12	☐	,3,4-triol	0.37	☐	☐	☐	☑	☐	☐	☑	☐	☐	☐	☐

Note: insert the relevant information and select only the techniques used in this study. In the empty columns you can insert any additional methods.

molecules

MDPI

Compound			Characterization											
Compound Nr.	New	Known (Ref. in main text)	Physical State (purity %)	1H NMR	13C NMR	2D NMR	HRMS	IR	XRD	GC/ HPLC				Data in SI
13	☐	butylamide	0.34	☐	☐	☐	☑	☐	☐	☑	☐	☐	☐	☐
14	☐	ndroside G	0.30	☐	☐	☐	☑	☐	☐	☑	☐	☐	☐	☐
15	☐	Sucrose	0.30	☐	☐	☐	☑	☐	☐	☑	☐	☐	☐	☐
16	☐	3'-en-3-ol	0.29	☐	☐	☐	☑	☐	☐	☑	☐	☐	☐	☐
17	☐	palmitate	0.21	☐	☐	☐	☑	☐	☐	☑	☐	☐	☐	☐
18	☐	Dendroxine	0.21	☐	☐	☐	☑	☐	☐	☑	☐	☐	☐	☐
19	☐	an-2-one	0.19	☐	☐	☐	☑	☐	☐	☑	☐	☐	☐	☐
20	☐	Dendrobine	0.18	☐	☐	☐	☑	☐	☐	☑	☐	☐	☐	☐
21	☐	tocopherol	0.11	☐	☐	☐	☑	☐	☐	☑	☐	☐	☐	☐
22	☐	Oleic acid	0.092	☐	☐	☐	☑	☐	☐	☑	☐	☐	☐	☐
23	☐	Nobilonine	0.077	☐	☐	☐	☑	☐	☐	☑	☐	☐	☐	☐
24	☐	phthalate	0.062	☐	☐	☐	☑	☐	☐	☑	☐	☐	☐	☐
25	☐	ndroside F	0.055	☐	☐	☐	☑	☐	☐	☑	☐	☐	☐	☐
26	☐	lenic acid	0.045	☐	☐	☐	☑	☐	☐	☑	☐	☐	☐	☐
27	☐	onobilin F	0.03	☐	☐	☐	☑	☐	☐	☑	☐	☐	☐	☐
	☐			☐	☐	☐	☐	☐	☐	☐	☐	☐	☐	☐

Compound			Characterization											
Compound Nr.	New	Known (Ref. in main text)	Physical State (purity %)	1H NMR	13C NMR	2D NMR	HRMS	IR	XRD	GC/ HPLC				Data in SI
	☐			☐	☐	☐	☐	☐	☐	☐	☐	☐	☐	☐
	☐			☐	☐	☐	☐	☐	☐	☐	☐	☐	☐	☐
	☐			☐	☐	☐	☐	☐	☐	☐	☐	☐	☐	☐
	☐			☐	☐	☐	☐	☐	☐	☐	☐	☐	☐	☐
	☐			☐	☐	☐	☐	☐	☐	☐	☐	☐	☐	☐
	☐			☐	☐	☐	☐	☐	☐	☐	☐	☐	☐	☐
	☐			☐	☐	☐	☐	☐	☐	☐	☐	☐	☐	☐
	☐			☐	☐	☐	☐	☐	☐	☐	☐	☐	☐	☐
	☐			☐	☐	☐	☐	☐	☐	☐	☐	☐	☐	☐
	☐			☐	☐	☐	☐	☐	☐	☐	☐	☐	☐	☐
	☐			☐	☐	☐	☐	☐	☐	☐	☐	☐	☐	☐
	☐			☐	☐	☐	☐	☐	☐	☐	☐	☐	☐	☐
	☐			☐	☐	☐	☐	☐	☐	☐	☐	☐	☐	☐
	☐			☐	☐	☐	☐	☐	☐	☐	☐	☐	☐	☐
	☐			☐	☐	☐	☐	☐	☐	☐	☐	☐	☐	☐
	☐			☐	☐	☐	☐	☐	☐	☐	☐	☐	☐	☐

molecules

MDPI

Compound			Characterization											
Compound Nr.	New	Known (Ref. in main text)	Physical State (purity %)	1H NMR	13C NMR	2D NMR	HRMS	IR	XRD	GC/ HPLC				Data in SI
	☐			☐	☐	☐	☐	☐	☐	☐	☐	☐	☐	☐
	☐			☐	☐	☐	☐	☐	☐	☐	☐	☐	☐	☐
	☐			☐	☐	☐	☐	☐	☐	☐	☐	☐	☐	☐
	☐			☐	☐	☐	☐	☐	☐	☐	☐	☐	☐	☐
	☐			☐	☐	☐	☐	☐	☐	☐	☐	☐	☐	☐
	☐			☐	☐	☐	☐	☐	☐	☐	☐	☐	☐	☐
	☐			☐	☐	☐	☐	☐	☐	☐	☐	☐	☐	☐
	☐			☐	☐	☐	☐	☐	☐	☐	☐	☐	☐	☐
	☐			☐	☐	☐	☐	☐	☐	☐	☐	☐	☐	☐
	☐			☐	☐	☐	☐	☐	☐	☐	☐	☐	☐	☐
	☐			☐	☐	☐	☐	☐	☐	☐	☐	☐	☐	☐
	☐			☐	☐	☐	☐	☐	☐	☐	☐	☐	☐	☐
	☐			☐	☐	☐	☐	☐	☐	☐	☐	☐	☐	☐
	☐			☐	☐	☐	☐	☐	☐	☐	☐	☐	☐	☐
	☐			☐	☐	☐	☐	☐	☐	☐	☐	☐	☐	☐
	☐			☐	☐	☐	☐	☐	☐	☐	☐	☐	☐	☐

molecules

MDPI

Compound			Characterization											
Compound Nr.	New	Known (Ref. in main text)	Physical State (purity %)	1H NMR	13C NMR	2D NMR	HRMS	IR	XRD	GC/ HPLC				Data in SI
	☐			☐	☐	☐	☐	☐	☐	☐	☐	☐	☐	☐
	☐			☐	☐	☐	☐	☐	☐	☐	☐	☐	☐	☐
	☐			☐	☐	☐	☐	☐	☐	☐	☐	☐	☐	☐
	☐			☐	☐	☐	☐	☐	☐	☐	☐	☐	☐	☐
	☐			☐	☐	☐	☐	☐	☐	☐	☐	☐	☐	☐
	☐			☐	☐	☐	☐	☐	☐	☐	☐	☐	☐	☐
	☐			☐	☐	☐	☐	☐	☐	☐	☐	☐	☐	☐
	☐			☐	☐	☐	☐	☐	☐	☐	☐	☐	☐	☐
	☐			☐	☐	☐	☐	☐	☐	☐	☐	☐	☐	☐
	☐			☐	☐	☐	☐	☐	☐	☐	☐	☐	☐	☐
	☐			☐	☐	☐	☐	☐	☐	☐	☐	☐	☐	☐
	☐			☐	☐	☐	☐	☐	☐	☐	☐	☐	☐	☐
	☐			☐	☐	☐	☐	☐	☐	☐	☐	☐	☐	☐
	☐			☐	☐	☐	☐	☐	☐	☐	☐	☐	☐	☐
	☐			☐	☐	☐	☐	☐	☐	☐	☐	☐	☐	☐
	☐			☐	☐	☐	☐	☐	☐	☐	☐	☐	☐	☐
