# Supplementary material for: Dendrobium Nobile Alcohol Extract Extends the Lifespan of Caenorhabditis elegans via hsf-1 and daf-16
Source: Molecules. 2024 Feb 19;29(4):908. doi: 10.3390/molecules29040908 (PMC10891841; doi:10.3390/molecules29040908)
Supplement: Supplementary file 1 [file molecules-29-00908-s001.zip › Supplementary Figure1-Mass spectrum of the compounds.pdf]

Mass spectrum of the compounds contained in DnAE

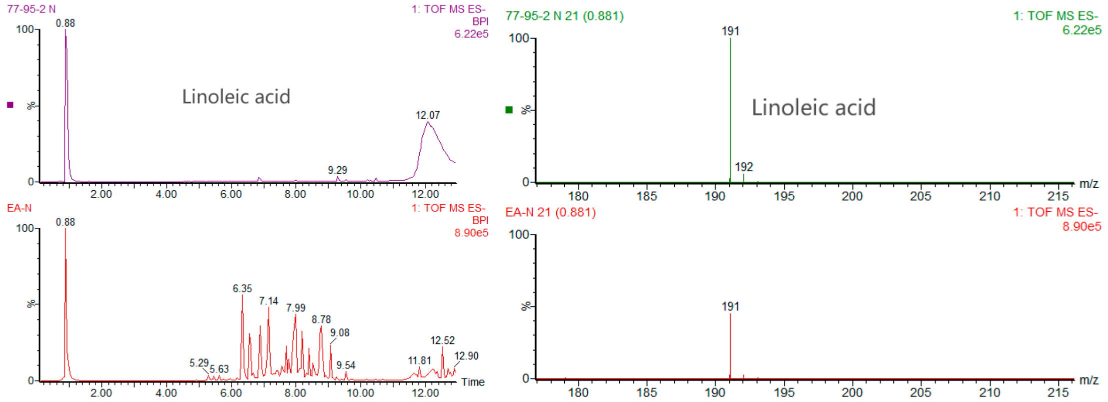

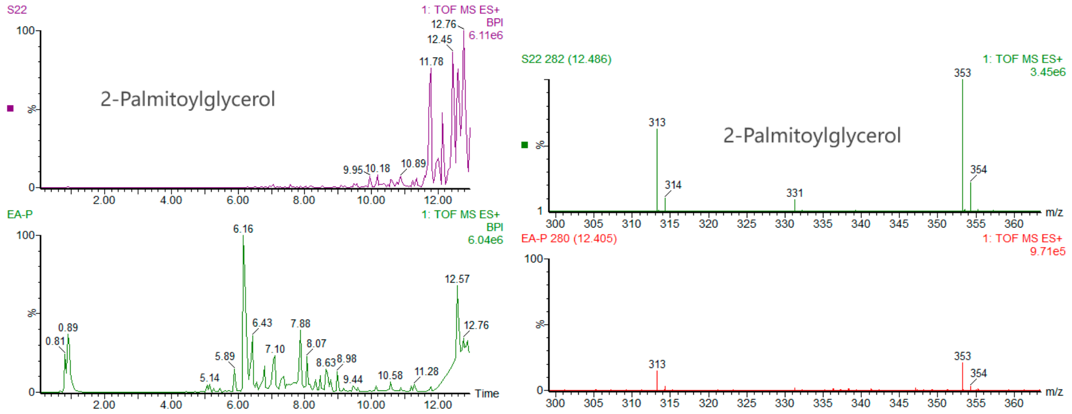

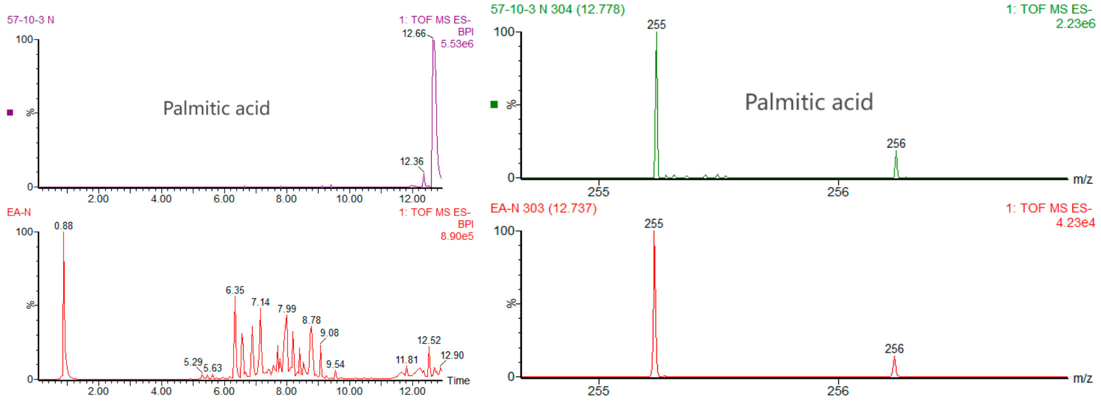

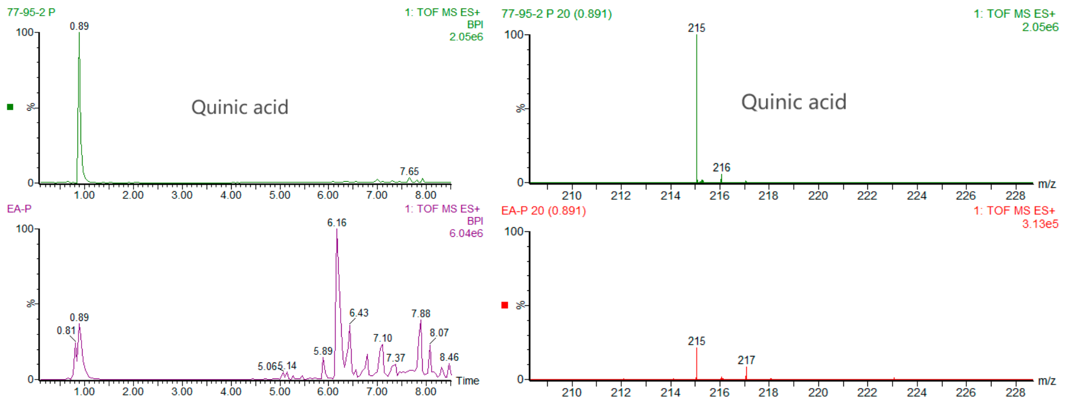

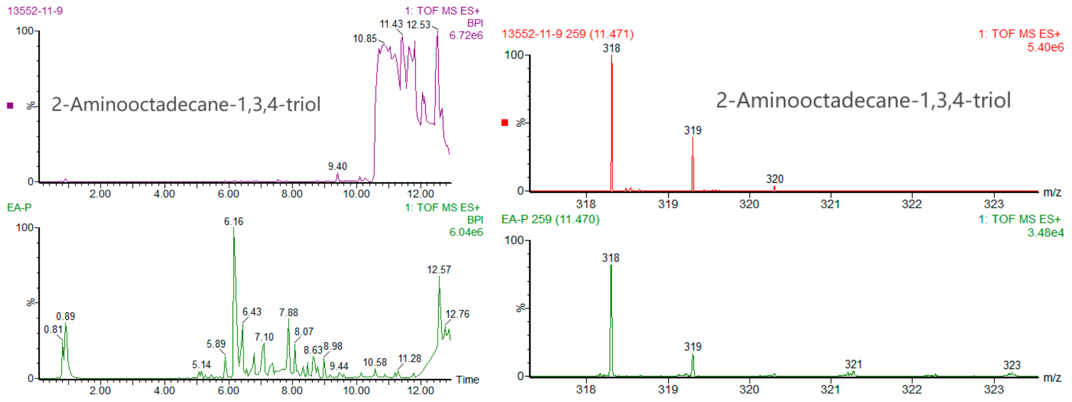
13552-11-9
1: TOF MS ES+
BPI
6.72e6
2-Aminooctadecane-1,3,4-triol
EA-P
1: TOF MS ES+
BPI
6.04e6
13552-11-9 259 (11.471)
1: TOF MS ES+
5.40e6
EA-P 259 (11.470)
1: TOF MS ES+
3.48e4
Time
m/z

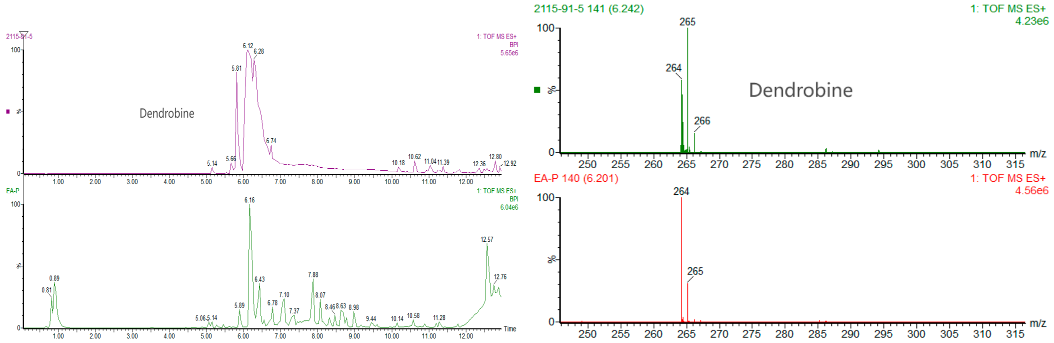
2115-91-5 141 (6.242)
1: TOF MS ES+
4.23e6
Dendrobine
EA-P 140 (6.201)
1: TOF MS ES+
4.56e6
m/z

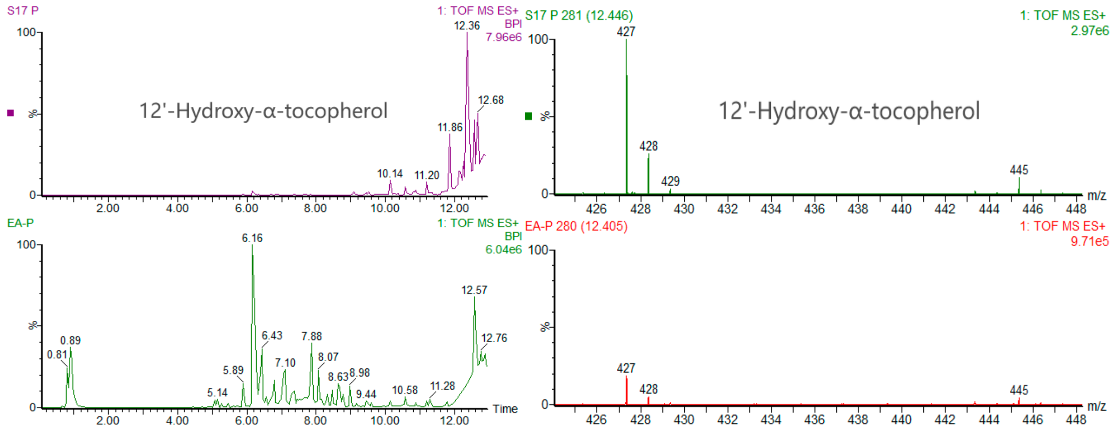
S17 P
1: TOF MS ES+
BPI
7.96e6
12'-Hydroxy-α-tocopherol
EA-P
1: TOF MS ES+
BPI
6.04e6
S17 P 281 (12.446)
1: TOF MS ES+
2.97e6
EA-P 280 (12.405)
1: TOF MS ES+
9.71e5
Time
m/z

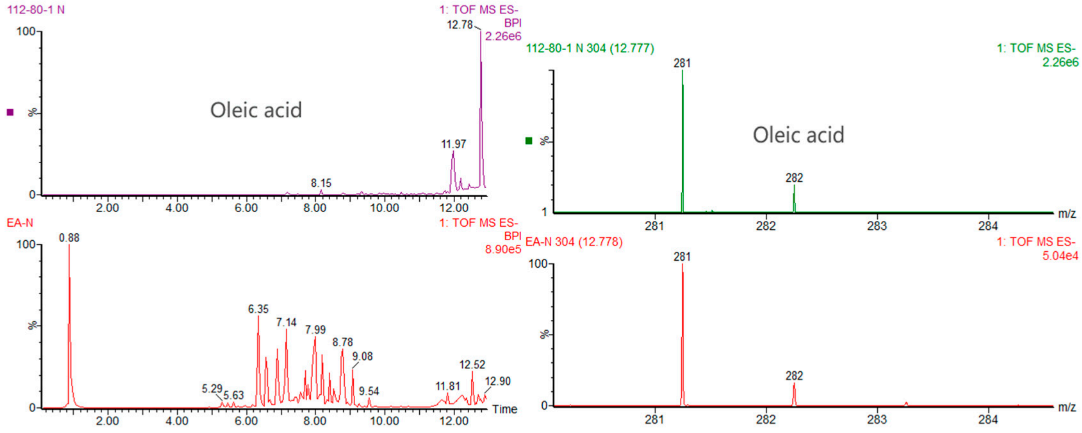
112-80-1 N
1: TOF MS ES-
BPI
2.26e6
Oleic acid
EA-N
1: TOF MS ES-
BPI
8.90e5
112-80-1 N 304 (12.777)
1: TOF MS ES-
2.26e6
EA-N 304 (12.778)
1: TOF MS ES-
5.04e4
Time
m/z

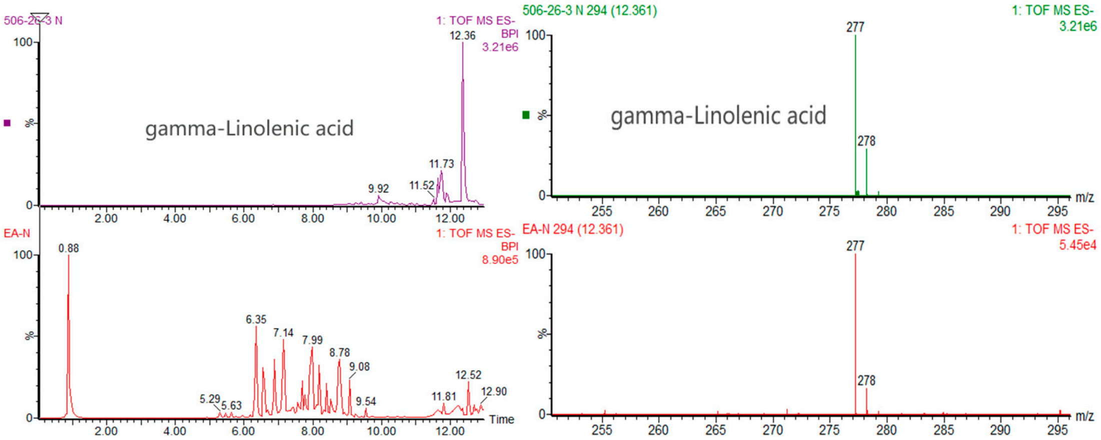
506-26-3 N
1: TOF MS ES-
BPI
3.21e6
gamma-Linolenic acid
12.36
11.73
11.52
9.92
EA-N
1: TOF MS ES-
BPI
8.90e5
0.88
6.35
7.14
7.99
8.78
9.08
9.54
5.29
5.63
11.81
12.52
12.90
Time
506-26-3 N 294 (12.361)
1: TOF MS ES-
3.21e6
gamma-Linolenic acid
277
278
m/z
EA-N 294 (12.361)
1: TOF MS ES-
5.45e4
277
278
m/z

Mass spectrum of the compounds not contained in DnAE

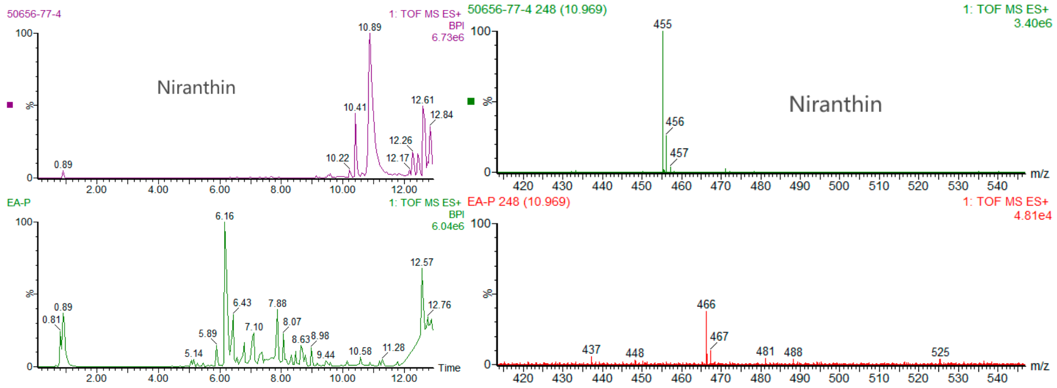

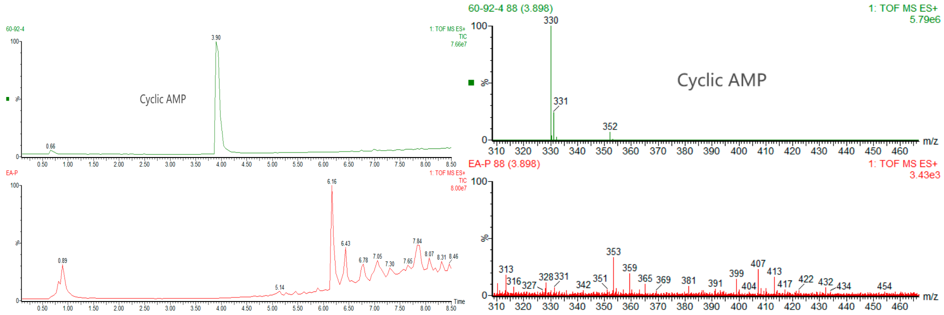

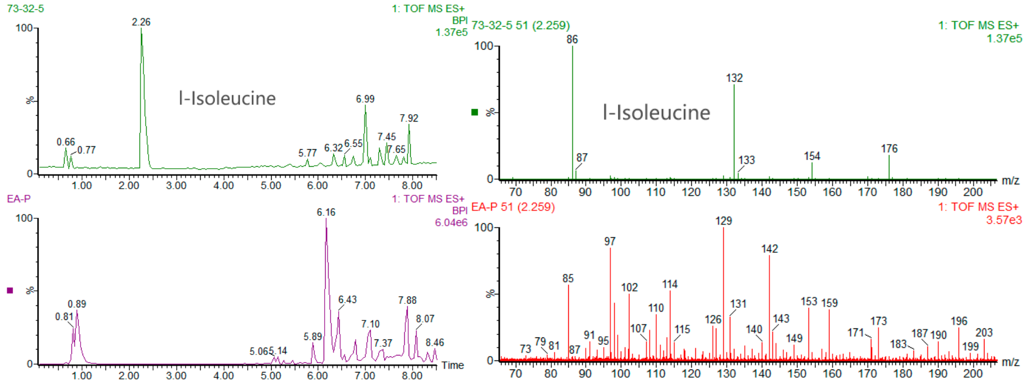

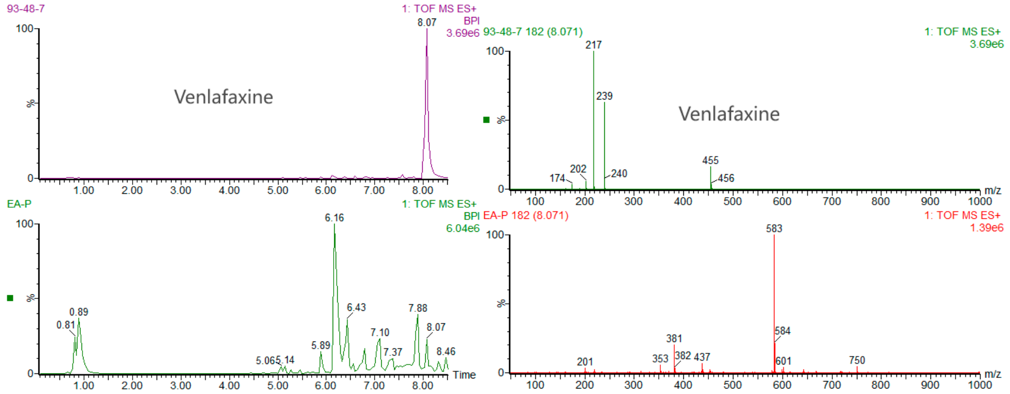

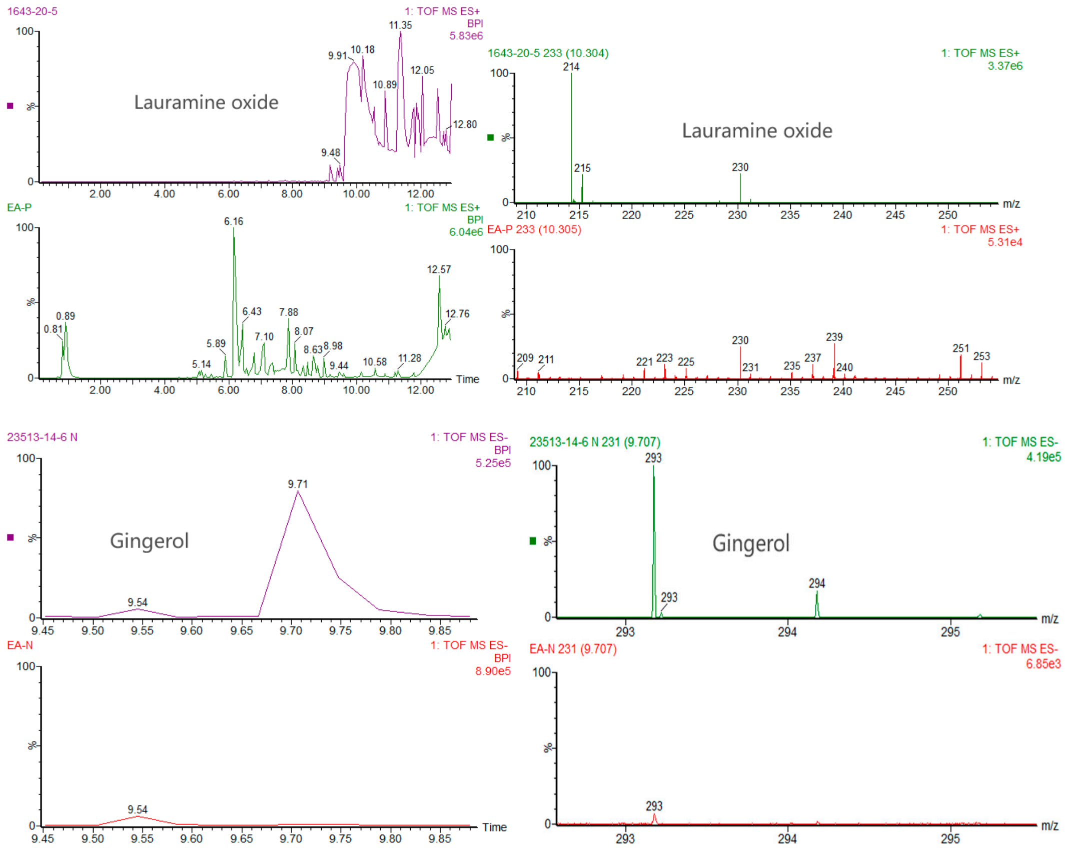
1643-20-5
1: TOF MS ES+
BPI
5.83e6
Lauramine oxide
EA-P
1: TOF MS ES+
BPI
6.04e6
Time
1643-20-5 233 (10.304)
1: TOF MS ES+
3.37e6
Lauramine oxide
EA-P 233 (10.305)
1: TOF MS ES+
5.31e4
m/z
23513-14-6 N
1: TOF MS ES-
BPI
5.25e5
Gingerol
EA-N
1: TOF MS ES-
BPI
8.90e5
Time
23513-14-6 N 231 (9.707)
1: TOF MS ES-
4.19e5
Gingerol
EA-N 231 (9.707)
1: TOF MS ES-
6.85e3
m/z
