# Supplementary material for: Dendrobium Nobile Alcohol Extract Extends the Lifespan of Caenorhabditis elegans via hsf-1 and daf-16
Source: Molecules. 2024 Feb 19;29(4):908. doi: 10.3390/molecules29040908 (PMC10891841; doi:10.3390/molecules29040908)
Supplement: Supplementary file 1 [file molecules-29-00908-s001.zip › Supplementary Tables.pdf]

Supplementary Tables

Contents:

Table S1. Lifespan of wild-type nematode (N2) treated with DnAE in different concentrations.

Table S2. Lifespan of wild-type nematode (N2) treated with 214 µmol/ml EtOH (consistent with the concentration of alcohol contained in 1 mg/ml DnAE)

Table S3 Effect of DnAE on oviposition rate of N2 worm.

Table S4. Effect of DnAE on pharyngeal pumping in wild-type nematode (N2).

Table S5. Effect of DnAE on body movement in wild-type nematode (N2).

Table S6. Effect of DnAE on lipofuscin in wild-type nematode (N2).

Table S7. Effect of DnAE on resistance to high temperature and oxidation in wild-type nematode (N2).

Table S8. Effect of DnAE on ROS level of N2 nematode.

Table S9. Effect of DnAE on SOD level of N2 nematode.

Table S10. Effect of DnAE on expression of HSP-12.6, SOD-3, GST-4, DAF-16 in mutant nematode.

Table S11. Effect of DnAE on lifespan of mutant nematode.

Table S12. Effects of DnAE on Parkinson's disease model.

Table S13. Effects of DnAE on Alzheimer's disease model.

Table S14. Effect of DnAE on gene expression at mRNA level in nematode.

Table S15. Primers used for the analysis of mRNA expression levels in nematode.

Table S1. Lifespan of wild-type nematode (N2) treated with DnAE in different concentrations.

**Figure 1A** Concentration		Control (0μM)	200μg/ml DnAE	500μg/ml DnAE	1000μg/ml DnAE
strain		**N2(WT)**	**N2(WT)**	**N2(WT)**	**N2(WT)**
Treatments		20°C/OP50 (dead)	20°C/OP50 (dead)	20°C/OP50 (dead)	20°C/OP50 (dead)
Mean±SEM	EXP.1	18.933±0.926	20.233±0.998	21.939±0.990	22.979±1.005
	EXP.2	18.663±0.728	20.374±0.972	21.243±0.961	22.270±0.938
	EXP.3	19.487±0.824	20.455±0.983	21.232±0.825	22.837±0.988
P value VS control	EXP.1		0.1594	0.0069	0.0013
	EXP.2		0.1325	0.0038	0.0009
	EXP.3		0.1932	0.0098	0.0023
N	EXP.1	63	62	65	68
	EXP.2	73	72	75	74
	EXP.3	73	73	77	77
change in mean lifespan	EXP.1		6.87%	15.88%	21.37%
	EXP.2		9.34%	14.01%	19.51%
	EXP.3		4.97%	8.95%	17.19%

Table S2. Lifespan of wild-type nematode (N2) treated with 214 μmol/ml EtOH (consistent with the concentration of alcohol contained in 1 mg/ml DnAE)

Figure	Strain	Treatment	Mean lifespan ±SEM (days)	P value VS control	change in mean lifespan	N
	**N2（WT）**	**20℃**	Days			
**1B**	EXP.1	control	18.643±0.672	0.4754	#	61
	EXP.1	214 μmol/ml EtOH	18.827±0.726			61
	EXP.2	control	19.224±0.526	0.2637	#	65
	EXP.2	214 μmol/ml EtOH	18.824±0.982			75
	EXP.3	control	19.453±0.530	0.6283	#	60
	EXP.3	214 μmol/ml EtOH	19.295±0.612			66

S1-S2: *p*-value was analyzed by log-rank (Mantel-Cox) test.
N: number of dead worms.
P <0.05 indicated that the experiment was statistically significant, while *P* >0.05 indicated that the experiment was not statistically significant.

Table S3 Effect of DnAE on oviposition rate of N2 worm.

	Days	Strains	Treatments	Mean offspring ±SEM	P value VS control	N
		**N2(WT)**	**OP50(dead) 20℃**			
	1	EXP.1	control	53.00±1.566	0.943	3
		EXP.1	1mg/ml DnAE	53.33±2.053		
	2	EXP.1	control	152.3±6.467	0.377	3
		EXP.1	1mg/ml DnAE	146.7±5.974		
	3	EXP.1	control	47.33±2.940	0.696	3
		EXP.1	1mg/ml DnAE	49.00±3.105		
	4	EXP.1	control	26.67±1.394	0.768	3
		EXP.1	1mg/ml DnAE	27.00±1.293		
	5	EXP.1	control	12.00±1.283	0.629	3
		EXP.1	1mg/ml DnAE	10.00±0.838		
	Total	EXP.1	control	291.3±6.323	0.169	3
		EXP.1	1mg/ml DnAE	282.7±6.923		
		**N2（WT）**	**OP50(dead) 20℃**			
	1	EXP.2	control	48.67±1.384	0.165	3
		EXP.2	1mg/ml DnAE	54.33±1.345		
	2	EXP.2	control	156.7±4.389	0.126	3
		EXP.2	1mg/ml DnAE	146.3±5.228		
	3	EXP.2	control	50.67±2.993	0.294	3
		EXP.2	1mg/ml DnAE	53.33±3.232		
	4	EXP.2	control	30.00±1.323	0.439	3
		EXP.2	1mg/ml DnAE	28.33±1.394		
	5	EXP.2	control	16.67±1.339	0.341	3
		EXP.2	1mg/ml DnAE	14.00±1.832		
	Total	EXP.2	control	302.7±6.822	0.578	3
**Figure1C**		EXP.2	1mg/ml DnAE	296.3±7.360		
		**N2（WT）**	**OP50(dead) 20℃**			
	1	EXP.3	control	49.33±2.283	0.493	3
		EXP.3	1mg/ml DnAE	46.33±1.92		
	2	EXP.3	control	142.3±4.263	0.916	3
		EXP.3	1mg/ml DnAE	142.7±3.911		
	3	EXP.3	control	49.00±1.293	0.922	3

	EXP.3	1mg/ml DnAE	49.33±1.178		
4	EXP.3	control	30.00±1.134	0.613	3
	EXP.3	1mg/ml DnAE	29.00±1.384		
5	EXP.3	control	12.67±0.490	0.402	3
	EXP.3	1mg/ml DnAE	10.33±0.379		
Total	EXP.3	control	283.3±6.915	0.346	3
	EXP.3	1mg/ml DnAE	277.7±6.559		

Table S4. Effect of DnAE on pharyngeal pumping in wild-type nematode (N2).

Figure	Strain	Treatment	Mean Pigment ±SEM	P value VS control	N
	**N2(WT)**	OP50(dead)	3 days of adult		
	EXP.1	20°C/control	29.30±0.6549	0.2326	20
	EXP.1	20°C/1mg/ml DnAE	29.65±0.6832		20
**1D**	EXP.2	20°C/control	31.37±0.6982	0.1937	20
	EXP.2	20°C/1mg/ml DnAE	30.63±0.6039		20
	EXP.3	20°C/control	31.16±0.6121	0.2142	20
	EXP.3	20°C/1mg/ml DnAE	30.43±0.5893		20

The pharyngeal pumping count time was 10 seconds per nematode. N was the experimental sample size, and P value was calculated by two-tailed t-test. P <0.05 indicated that the experiment was statistically significant

Table S5. Effect of DnAE on body movement in wild-type nematode (N2).

Figure	Strain	Treatment	Mean Pigment ±SEM	P value VS control	N
	**N2(WT)**	OP50(dead)	3 days of adult		
	EXP.1	20°C/control	34.70±0.7749	0.2888	20
	EXP.1	20°C/1mg/ml DnAE	35.65±0.6893		20
**1E**	EXP.2	20°C/control	32.46±0.5452	0.1733	20
	EXP.2	20°C/1mg/ml DnAE	34.13±0.5029		20
	EXP.3	20°C/control	31.16±0.4832	0.364	20
	EXP.3	20°C/1mg/ml DnAE	32.17±0.5923		20

The body movement count time was 20 seconds per nematode. N was the experimental sample size, and P value was calculated by two-tailed t-test. P <0.05 indicated that the experiment was statistically significant

Table S6. Effect of DnAE on lipofuscin in wild-type nematode (N2).

Figure	Strain	Treatments	Mean Pigment ±SEM	*P* value VS control	N
	**N2(WT)**	**20℃ 5 days**			
	EXP.1	control	18.06±0.921	<0.00001	29
	EXP.1	1mg/ml DnAE	8.635±0.448		30
	EXP.2	control	16.31±0.954	<0.00001	30
**1F**	EXP.2	1mg/ml DnAE	7.41±0.351		30
	EXP.3	control	18.39±0.720	<0.00001	25
	EXP.3	1mg/ml DnAE	10.02±0.322		29
	**N2(WT)**	**20℃ 10 days**			
	EXP.1	control	36.54±1.293	0.0011	29
		1mg/ml DnAE	33.44±1.048		30
**1F**	EXP.2	control	34.31±0.954	0.0008	30
		1mg/ml DnAE	30.41±1.151		30
	EXP.3	control	29.89±0.820	0.0015	25
		1mg/ml DnAE	27.02±0.769		29

On the 5 and 10 day of the adult worm, the accumulation of lipofuscin in intestinal tissues of the treated and untreated nematodes was photographed and counted. Fluorescence intensity was analyzed by Image J, and *p* value was calculated by two-tailed t-test, where $P < 0.05$ indicated that the experiment was statistically significant

Table S7. Effect of DnAE on resistance to high temperature and oxidation in wild-type nematode (N2).

Figure	Strain	Treatment	Mean lifespan ±SEM	P value VS control	change in mean lifespan	N
	**N2**	**Paraquat**	Days			
4B	EXP.1	20°C/control	9.436±0.493	0.010	21.87%	80
	EXP.1	20°C/1mg/ml DnAE	11.500±0.611			82
	EXP.2	20°C/control	8.581±0.343	0.031	19.24%	70
	EXP.2	20°C/1mg/ml DnAE	10.232±0.549			76
	EXP.3	20°C/control	9.059±0.603	0.018	19.87%	80
	EXP.3	20°C/1mg/ml DnAE	10.858±0.307			85
	**N2**	**H_2O_2**	Hours			
4A	EXP.1	20°C/control	4.750±0.234	0.007	23.16%	74
	EXP.1	20°C/1mg/ml DnAE	5.850±0.311			78
	EXP.2	20°C/control	4.612±0.243	0.002	19.39%	84
	EXP.2	20°C/1mg/ml DnAE	5.506±0.303			80
	EXP.3	20°C/control	4.085±0.206	0.011	20.38%	85
	EXP.3	20°C/1mg/ml DnAE	4.917±0.278			81
	**N2**	**37°C**	Hours			
3A	EXP.1	control	3.256±0.200	0.0007	34.09%	65
	EXP.1	1mg/ml DnAE	4.366±0.254			78
	EXP.2	control	3.612±0.173	0.0011	28.39%	88
	EXP.2	1mg/ml DnAE	4.637±0.209			81
	EXP.3	control	3.485±0.201	0.0021	30.78%	80
	EXP.3	1mg/ml DnAE	4.558±0.208			81

p-value was analyzed by log-rank (Mantel-Cox) test.

N: number of dead worms.

P <0.05 indicated that the experiment was statistically significant, while *P* >0.05 indicated that the experiment was not statistically significant.

Table S8. Effect of DnAE on ROS level of N2 nematode.

Figure	Strain	Treatment	Mean ± SEM	P value VS Control	N
**4D**	**N2(WT)**	20℃ OP50(dead)			
	EXP.1	Control	10.24±0.1531		22
	EXP.1	1mg/ml DnAE	7.889±0.2475	<0.0001	23
	EXP.1	4mM Paraquat	18.23±0.2392	<0.0001	20
	EXP.1	4mM Paraquat 1mg/ml DnAE	16.40±0..4523	<0.0001	21
	EXP.2	Control	14.318±0.521		33
	EXP.2	1mg/ml DnAE	10.964±0.391	<0.0001	29
	EXP.2	4mM Paraquat	20.140±0.620	<0.0001	33
	EXP.2	4mM Paraquat 1mg/ml DnAE	18.172±0.667	<0.0001	35
	EXP.3	Control	18.283±0.226		30
	EXP.3	1mg/ml DnAE	15.293±0.469	<0.0001	27
	EXP.3	4mM Paraquat	27.394±0.392	<0.0001	30
	EXP.3	4mM Paraquat 1mg/ml DnAE	22.312±0.683	<0.0001	33

ROS levels were quantified using the cell membrane-permeable reactive oxygen species (ROS) detection probe H2DCFH-DA.the accumulation of ROS was photographed and counted. Fluorescence intensity was analyzed by Image J, and *p* value was calculated by two-tailed t-test, where P <0.05 indicated that the experiment was statistically significant.

Table S9. Effect of DnAE on SOD level of N2 nematode.

Figure	Strain	Treatment	SOD activity	P value VS control
	**N2（WT）**	**20℃**		
**4C**	EXP.1	control	7.3321±0.122	0.0050
	EXP.1	1mg/ml DnAE	9.7259±0.096	
	EXP.2	control	6.224±0.106	0.0037
	EXP.2	1mg/ml DnAE	10.824±0.192	
	EXP.3	control	12.453±0.249	0.0183
	EXP.3	1mg/ml DnAE	14.295±0.212	

p value was calculated by two-tailed t-test

N: number of dead worms. N⩾1000.

P <0.05 indicated that the experiment was statistically significant.

Table S10. Effect of DnAE on expression of HSP-12.6, SOD-3, GST-4, DAF-16 in mutant nematode.

Figure	Strain	Treatment	Fluorescence intensity Mean ± SEM	P valuel	N
	**TJ375** ***gpIs1 (hsp-16.2p::GFP)***	OP50(dead)			
3C	EXP.1	20℃/control	4.614±0.2891	0.0021	31
	EXP.1	20℃/1mg/ml DnAE	6.457±0.6876		31
	EXP.1	35℃ 1h	26.44±3.199	0.0249	25
	EXP.1	35℃1h/1mg/ml DnAE	28.02±2.548		27
	EXP.2	20℃/control	5.233±0.158	0.0036	36
	EXP.2	20℃/1mg/ml DnAE	7.526±0.152		34
	EXP.2	35℃ 1h	22.26±2.293	0.0162	34
	EXP.2	35℃1h/1mg/ml DnAE	24.02±2.521		27
	EXP.3	20℃/control	4.238±0.1928	0.0022	28
	EXP.3	20℃/1mg/ml DnAE	6.263±0.5278		32
	EXP.3	35℃ 1h	25.27±2.349	0.0527	22
	EXP.3	35℃1h/1mg/ml DnAE	28.32±2.364		25
	**CL2166** ***dvIs19*** **(gst-4p::GFP)**	20℃ OP50(dead)			
5B	EXP.1	control	14.50±0.4102	<0.0001	38
	EXP.1	1mg/ml DnAE	19.11±0.4200		36
	EXP.1	4mM Paraquat	9.581±0.3027	<0.0001	43
	EXP.1	1mg/ml DnAE 4mM Paraquat	12.22±0.3090		39
	EXP.2	control	11.26±0.3760	<0.0001	34
	EXP.2	1mg/ml DnAE	14.77±0.3224		36
	EXP.2	4mM Paraquat	8.283±0.3983	<0.0001	35
	EXP.2	1mg/ml DnAE 4mM Paraquat	10.27±0.3451		33
	EXP.3	control	9.553±0.3964	<0.0001	31
	EXP.3	1mg/ml DnAE	13.28±0.4340		36
	EXP.3	4mM Paraquat	8.391±0.3237	<0.0001	40
	EXP.3	1mg/ml DnAE 4mM Paraquat	10.25±0.3543		38
**5C**	**CF1553** ***zcIs13*** **(sod-3p::GFP)**	OP50(dead)			

EXP.1	control	11.00±0.2183	<0.0001	21
EXP.1	1mg/ml DnAE	13.21±0.2854		24
EXP.1	4mM Paraquat	8.163±0.2220	0.0003	32
EXP.1	1mg/ml DnAE 4mM Paraquat	9.577±0.2282		23
EXP.2	control	12.37±0.2233	<0.0001	32
EXP.2	1mg/ml DnAE	14.22±0.2804		25
EXP.2	4mM Paraquat	8.273±0.2660	0.0023	32
EXP.2	1mg/ml DnAE 4mM Paraquat	9.746±0.2228		27
EXP.3	control	10.88±0.2142	0.0003	33
EXP.3	1mg/ml DnAE	12.54±0.2464		34
EXP.3	4mM Paraquat	8.453±0.2022	0.0010	32
EXP.3	1mg/ml DnAE 4mM Paraquat	9.863±0.3100		32

Figure	Strain	Treatment	Cytosolic	Intermediate	Nuclear	N
	**TJ356 zIs356(daf-16p::daf-16a/b::GFP)**	20°C OP50(dead)				
	EXP.1	control	35			35
	EXP.1	1mg/ml DnAE	29			29
	EXP.1	4mM Paraquat	3	18	12	33
	EXP.1	4mM Paraquat 1mg/ml DnAE	2	11	14	27
**5A**	EXP.1	control	30			30
	EXP.1	1mg/ml DnAE	26			26
	EXP.1	4mM Paraquat	3	20	7	30
	EXP.1	4mM Paraquat 1mg/ml DnAE	3	12	16	31
	EXP.1	control	25			25
	EXP.1	1mg/ml DnAE	26			26
	EXP.1	4mM Paraquat	3	15	6	24
	EXP.1	4mM Paraquat 1mg/ml DnAE	2	10	12	24

Fluorescence intensity was analyzed by Image J, and p value was calculated by two-tailed t-test, where P <0.05 indicated that the experiment was statistically significant.

Table S11. Effect of DnAE on lifespan of mutant nematode.

Figure	Strain	Treatment	Mean lifespan ±SEM (days)	P value VS control	change in mean lifespan	N
	**PS3551** ***hsf-1 (sy441).***	20°C OP50(dead)	Days			
3D	EXP.1	control	15.172±0.926	0.2559	#	60
	EXP.1	1mg/ml DnAE	15.688±1.298			77
	EXP.2	control	15.224±0.689	0.2862	#	77
	EXP.2	1mg/ml DnAE	14.824±0.982			85
	EXP.3	control	14.453±0.536	0.6161	#	66
	EXP.3	1mg/ml DnAE	14.075±0.619			68
	**CF1038** ***daf-16 (mu86).***	20°C OP50(dead)				
6B	EXP.1	control	12.361±0.564	0.1651	#	79
	EXP.1	1mg/ml DnAE	12.727±0.588			78
	EXP.2	control	13.158±0.511	0.2112	#	70
	EXP.2	1mg/ml DnAE	12.215±0.405			80
	EXP.3	control	11.832±0.505	0.2938	#	61
	EXP.3	1mg/ml DnAE	12.292±0.650			64
	**CB1370** ***daf-2 (e1370).***	20°C OP50(dead)				
6C	EXP.1	control	43.410±1.886	0.1373	#	77
	EXP.1	1mg/ml DnAE	42.788±2.620			73
	EXP.2	control	42.767±1.458	0.110	#	88
	EXP.2	1mg/ml DnAE	43.157±1.520			78
	EXP.3	control	39.284±1.592	0.716	#	67
	EXP.3	1mg/ml DnAE	40.032±1.532			76

p-value was analyzed by log-rank (Mantel-Cox) test.

N: number of dead worms.

P <0.05 indicated that the experiment was statistically significant, while *P* >0.05 indicated that the experiment was not statistically significant.

Table S12. Effects of DnAE on Parkinson

PD					
Figure	Strain	Treatment	Mean±SEM Days	P value	N
7B	**BZ555** ***egIs1*****(DAT-1::GFP)**	20°C OP50(dead)			
	EXP.1	50mM 6-OHDA	16.10±0.7446		22
	EXP.1	50mM 6-OHDA +1mg/ml DnAE	20.20±0.7072	<0.0001	20
	EXP.1	50mM 6-OHDA +2mM L-DA	21.09±0.5886	<0.0001	20
	EXP.1	Control	22.04±0.5842	<0.0001	20
	EXP.2	50mM 6-OHDA	15.04±0.4292		23
	EXP.2	50mM 6-OHDA +1mg/ml DnAE	18.31±0.8639	0.0036	22
	EXP.2	50mM 6-OHDA +2mM L-DA	20.12±0.5639	<0.0001	22
	EXP.2	Control	20.93±0.9378	<0.0001	21
	EXP.3	50mM 6-OHDA	16.32±0.5386		25
	EXP.3	50mM 6-OHDA +1mg/ml DnAE	18.54±0.7210	0.0004	24
	EXP.3	50mM 6-OHDA +2mM L-DA	19.38±0.5248	<0.0001	29
	EXP.3	Control	21.22±0.9218	<0.0001	21
7A	**N5901** **Punc-54::α-syn::YFP**	**5 days of adult**			
	EXP.1	control	11.230±1.073	<0.0001	35
	EXP.1	1mg/ml DnAE	9.188±1.534		27
	EXP.1	DR	8.347±1.069	<0.0001	29
	EXP.1	DR+1mg/ml DnAE	6.420±1.070		32
	EXP.2	control	14.678±1.580	0.0008	30
	EXP.2	1mg/ml DnAE	11.391±1.447		31
	EXP.2	DR	9.347±1.273	0.0002	26
	EXP.2	DR+1mg/ml DnAE	7.620±1.270		34
	EXP.3	control	13.348±1.720	0.0021	27
	EXP.3	1mg/ml DnAE	11.321±1.441		27
	EXP.3	DR	10.233±1.772	<0.0001	28
	EXP.3	DR+1mg/ml DnAE	8.273±1.491		30

Fluorescence intensity was analyzed by Image J, and p value was calculated by two-tailed t-test, where P <0.05 indicated that the experiment was statistically significant.

Table S13. Effects of DnAE on Alzheimer

Figure	Strain	Treatment	Mean hours ±SEM	P value VS control	change in mean time	N
**7C**	**CL4176 dvIs27 [myo-3p::A-Beta (1-42)::let-851 3'UTR)]**	**25°C**	Hours			
	EXP.1	control	32.967±0.283			61
	EXP.1	DnAE	34.328±0.344	0.0023	4.13%	61
	EXP.2	DR	34.355±0.367	0.0019	4.21%	65
	EXP.2	DR + DnAE	35.123±0.345	<0.0001	6.53%	73
	EXP.2	control	30.453±0.330			60
	EXP.2	DnAE	32.495±0.322	0.0033	6.70%	66
	EXP.2	DR	32.667±0.427	0.0024	7.27%	67
	EXP.2	DR + DnAE	33.371±0.571	0.0002	9.66%	77
	EXP.3	control	32.189±0.327			70
	EXP.3	DnAE	34.501±0.401	0.0043	7.18%	71
	EXP.3	DR	34.428±0.390	0.0053	7.01%	68
	EXP.3	DR + DnAE	35.652±0.326	0.0004	10.76%	71

p-value was analyzed by log-rank (Mantel-Cox) test.

N: number of dead worms.

P <0.05 indicated that the experiment was statistically significant, while P >0.05 indicated that the experiment was not statistically significant.

Table S14. Effect of DnAE on gene expression at mRNA level in nematode.

Gene	EXP.1	EXP.2	EXP.3	Mean	SEM
Control	1	1	1	1	0
daf-16	2.329	3.577	2.345	2.884	0.366
skn-1	0.763	0.969	1.063	0.932	0.088
daf-2	0.491	0.680	0.562	0.578	0.055
gst-4	1.507	1.780	2.275	1.854	0.224
sod-3	2.175	2.051	1.931	2.053	0.070
ctl-1	1.259	1.456	1.672	1.462	0.119
hsf-1	4.665	3.164	3.498	3.775	0.454
hsp-16.1	1.886	1.411	1.567	1.621	0.139
hsp-16.2	1.264	1.453	1.483	1.400	0.068
hsp-6	1.820	1.759	1.881	1.820	0.035
hsp-60	1.417	1.133	1.283	1.278	0.081
hsp-12.6	2.965	3.132	2.893	2.997	0.070

Table S14. Primers used for the analysis of mRNA expression levels in nematode.

Gene	Forward primer	Reverse primer
cdc-42	CTGCTGGACAGGAAGATTACG	CTCGGACATTCTCGAATGAAG
hsp-16.2	CTGCAGAATCTCTCCATCTGAGTC	AGATTCGAAGCAACTGCACC
hsp-60	AGGAGAAGCTTAATGAGCG	ACACGGTCCTTCTTCTCT
hsp-6	AGGAACAACAGAGTAAGATTTTC	TCGATTTGGTCCTTGGAAAG
sod-3	AGCATCATGCCACCTACGTGA	CACCACCATTGAATTTCAGCG
ctl-1	GAATGTGAAGAATTATTTCGCTGA	GAATGTGAAGAATTATTTCGCTGA
daf-2	CGGTGCGAAGAGAGGATATT	TACAGAGGTCGCCGTTACTG
gst-4	TCCGTCAATTCACTTCTTCCG	AAGAAATCATCACGGGCTGG
hsf-1	TTGACGACGACAAGCTTCCAGT	AAAGCTTGCACCAGAATCATCCC
daf-16	TTTCCG TCC CCG AACTCA	ATTCGCCAACCCATGATGG
hsp-12.6	GTG ATG GCTGACGAAGGAAC	GGGAGGAAGTTATGGGCTTC
skn-1	AGTGTCGGCGTTCCAGATTTC	GTCGACGAATCTTGCGAATCA
hsp-16.1	GTCACTTTACCACTATTTCCGTCCAGC TCAACGTTC	CAACGGGCGCTTGCTGAATTGGAATA GATCTTCC
